# Generation of carbonated peridotite melts via biogenic sediment recycling in modern subduction zones

**DOI:** 10.1126/sciadv.aec1599

**Published:** 2026-05-06

**Authors:** Carlos Errázuriz-Henao, Arturo Gómez-Tuena, Marion Weber, Mattia Parolari, Berengere Mougel, Marine Paquet, Eric Hellebrand, Oliver Plümper, Paul Mason, Frédéric Moynier

**Affiliations:** ^1^ Departamento de Geociencias y Medio Ambiente, Facultad de Minas, Universidad Nacional de Colombia, Carrera 80 No. 65-223, Medellín, Colombia.; ^2^ Instituto de Geología, Universidad Nacional Autónoma de México, Ciudad Universitaria, 04510 Coyoacán, CDMX, Mexico.; ^3^ Laboratorio Nacional de Geoquímica y Mineralogía, Universidad Nacional Autónoma de México, Ciudad Universitaria, 04510 Coyoacán, CDMX, Mexico.; ^4^ Instituto de Geociencias, UNAM, Blvd. Juriquilla 3001, 76230 Juriquilla, Querétaro, Mexico.; ^5^ Université de Lorraine, CNRS, Centre de Recherches Pétrographiques et Géochimiques, Nancy F-54000, France.; ^6^ Department of Earth Sciences, Utrecht University, Princetonlaan 8a, 3584 CB Utrecht, Netherlands.; ^7^ Faculty of Geosciences and MARUM–Center for Marine Environmental Sciences, University of Bremen, Klagenfurter Straße 2-4, 28359 Bremen, Germany.; ^8^ Université Paris Cité, Institut de Physique du Globe de Paris, CNRS, Paris 75005, France.

## Abstract

The subduction of carbonate-rich biogenic sediments influences Earth’s carbon cycle and regulates long-term climate. Yet, the processes controlling their behavior at the subduction interface remain debated. Here, we investigate the role of carbonate subduction through the petrogenesis of Quaternary rear-arc monogenetic volcanoes in Colombia. These volcanoes display unusual compositions resembling intraplate carbonated peridotite melts and experimental melts from peridotite + CO_2_ systems. Their geochemistry reflects an anomalous mantle source modified by subducted sediments from the Panama Basin, a large-scale region of high primary productivity and carbonate accumulation. However, pristine subducted sediments alone cannot explain the full compositional range of rear-arc Colombian lavas. Instead, we propose that sediment partial melting beneath the arc front produces carbonate- and apatite-rich restites that act as a recycled carbonated component, contributing to mantle chemical heterogeneity. This model helps explain the geochemical diversity of mantle-derived intraplate lavas and establishes a direct link between biogeochemical cycles, subduction, and the evolution of the solid Earth.

## Teaser

Volcanoes reveal how subducted marine carbonates transfer Earth’s surface biogeochemical signatures to the convecting mantle.

## INTRODUCTION

Subduction zones play a major role in balancing the surface and deep carbon cycles (*1*). This ongoing process regulates global climate over geological timescales (*2*) and modulates the composition and evolution of Earth’s mantle (*3*). However, the mechanisms that transport and modify carbonate-rich sediments during subduction, and the timescales at which carbon is redistributed among planetary reservoirs, remain subjects of debate. Current experimental, numerical, and petrological evidence suggests that ~27 to 55% of subducted carbon is recycled back to the atmosphere through partial melting and/or decarbonation of carbonate-rich lithologies (*4*–*6*). In contrast, some intraplate magmas, such as kimberlites and nephelinites, as well as plume-related high-μ (HIMU)–type lavas, are believed to originate from carbonated peridotite sources (*7*–*10*), implying that a substantial fraction of the remaining subducted carbon is transported to deeper regions of the convecting mantle.

The difficulty in tracking carbon fluxes among terrestrial reservoirs stems from its complex partitioning into sediments, fluids, gases, melts, and the biosphere, as well as by the analytical challenges in obtaining precise and accurate measurements of its content in magmatic and mantle rocks (*5*). Over the past decade, stable zinc (Zn) isotopes have become essential tracers of subducted carbon, owing to the large isotopic differences between silicic and carbonated components (*11*). Among subducted materials, carbonates are the most enriched in heavy Zn isotopes. Recent studies have reported heavy Zn isotopic compositions in HIMU-type ocean island basalts (OIBs) and sodic nephelinites from Eastern China, suggesting the presence of deeply stored carbonated materials in the mantle beneath cratons and oceanic basins (*10*, *12*). Nonetheless, deciphering the nature of their carbon-rich sediment precursors and their transformations along the subduction channel remains challenging. Unlike subduction-related magmas, intraplate rocks are not directly linked to active convergent margins but instead originate from mantle domains with a complex and ancient metasomatic history. In this context, subduction-related magmatism provides the most direct means for tracing subducted components back to trench sedimentary sequences and for recognizing the potential influence of carbonate subduction on mantle evolution. Oceanographic and tectonic data indicate that substantial carbonate subduction (>5% CaCO_3_) occurs mainly within select active subduction zones globally, particularly along west-facing equatorial margins with high primary productivity, including Central America, the Northern Andes, and Peru (*13*). However, identifying the specific processes that allow these carbonates to survive subduction and enter the deeper mantle is still puzzling, as a definitive carbonate signature has yet to be recognized in mantle-derived magmas from these regions.

Here, we present geochemical data, including major and trace elements, Sr-Nd-Pb-Hf radiogenic isotopes, and Zn stable isotopes, from a unique suite of Quaternary basanite-nephelinites from Colombia. These rocks resemble carbonated peridotite melts from intraplate settings (*12*) and are consistent with experimental peridotite + CO_2_ melt datasets (*14*). These lavas represent a previously unrecognized volcanic suite type within the Andean Cordillera. They erupted in the rear-arc region (~70 km behind the arc-front) of the Colombian subduction zone, where the tectonic, biological, and oceanographic conditions of the Panama Basin (PB) have fostered some of the highest biogenic carbonate concentrations (~7 CaCO_3_ wt %) in modern trench sediments (*15*–*17*). Because carbonated components are not expected to melt at sub-arc conditions (*18*), rear-arc magmas such as these offer a window into the fate of carbonate at greater mantle depths. To better trace these carbonated components into the mantle, we also conducted Zn isotopic analyses of deep-sea sediments from the PB. Our results reveal a close compositional link between the mantle-derived rocks and recently subducted carbonate-rich sediments, providing a modern analog for the involvement of carbonated components known to reside deep in Earth’s mantle.

## RESULTS AND DISCUSSION

### The Colombian subduction system

**Fig. 1.**
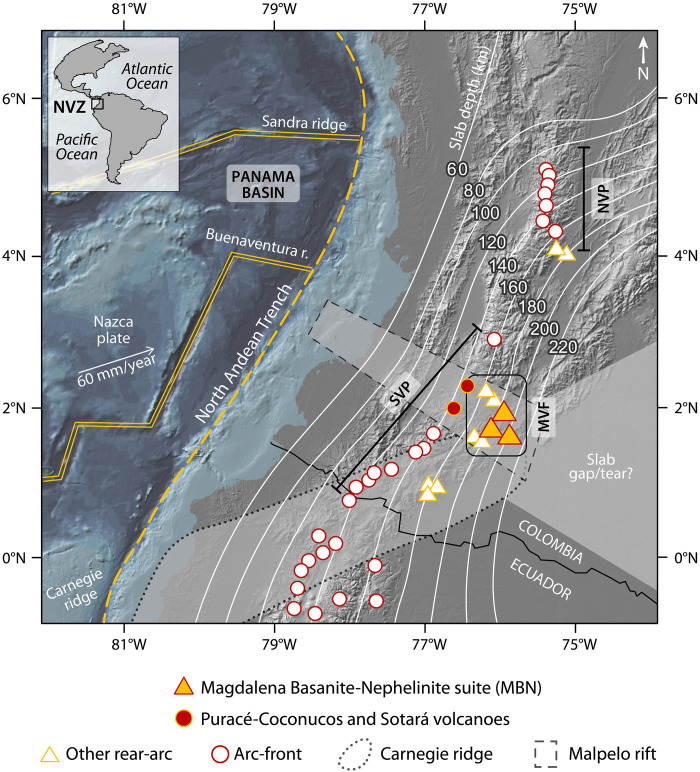
Map of the Northern Colombian Andes. Main tectonic features of the PB, slab depth contours (*101*), projections of subducted ridges, and location of the main arc-front volcanoes and rear-arc volcanic clusters. For a detailed map of the study area and sample location, please refer to fig. S1. NVZ, North Volcanic Zone; MVF, Magdalena Volcanic Field; SVP, South Volcanic Province; NVP, North Volcanic Province.

The North Andean arc was formed by the relatively fast (~60 mm/year) subduction of the young Nazca plate [12 to 20 million years ago (Ma)] beneath the South American Plate (*19*) (Fig. 1). Its configuration is the result of the subduction of multiple ridges and extinct rifts that developed from the complex tectonic history of the PB (*20*, *21*). To the north, subduction of the Sandra Ridge coincides with the development of the Colombian flat-slab, which restricts the northward extent of arc-front volcanism. To the south, the high-angle subduction of the Buenaventura Ridge creates a volcanic gap and marks the northern boundary of the South Volcanic Province that continues uninterrupted into Ecuador (Fig. 1). In this study, we focus on the Magdalena Volcanic Field (MVF), situated in the rear-arc region of the South Volcanic Province, ~70 km east of the active andesitic volcanoes of Puracé-Coconucos and Sotará (see fig. S1). The MVF is the largest of Colombia’s three rear-arc monogenetic volcanic fields, spanning ~2000 km^2^ and comprising around 30 volcanic edifices (*22*) (Fig. 1). Local variations in regional gravity anomalies, mantle seismic anisotropy, and broad changes in teleseismic tomography indicate that the MVF is situated directly above a major change in the geometry of the subducted Nazca plate (*23*, *24*). This anomaly likely reflects the subduction of the Malpelo Rift and Carnegie Ridge at depth, as well as the development of a possible slab gap/tear in the Nazca plate below ~200 km (*24*), closely corresponding to the projected slab depth beneath the MVF (Fig. 1).

**Fig. 2.**
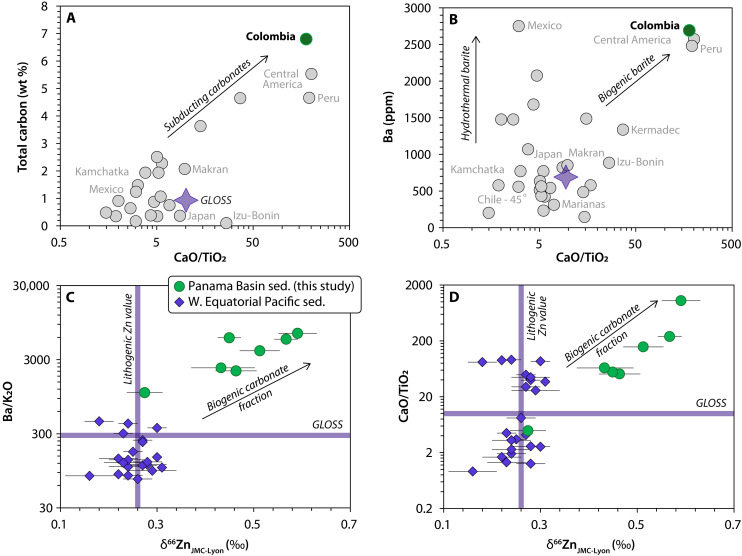
Carbon, major, trace, and Zn isotopic composition of PB sediments and GLOSS. (**A** and **B**) Average composition of subducting sediments from multiple trenches and average GLOSS showing the carbon-rich and Ba-rich nature of Colombian trench sediments. All major and trace element data in (A) and (B) are from ref. (*13*), whereas carbon contents are from ref. (*15*). ppm, parts per million. (**C** and **D**) Relationship between the Zn isotopic composition and the contribution from biogenic carbonate and barite of East Equatorial Pacific sediments from the PB (this study) and West Equatorial Pacific sediments from the south China Sea (*11*). Major and trace elements from individual PB samples are from ref. (*21*).

The nonerosive character of the Colombian subduction zone (*25*) implies that the subducted components originate mainly from the Nazca oceanic plate and the overlying oceanic sediments deposited in the PB. Deep-sea drilling cores from the PB (DSDP sites 84 and 504) reveal an ~300-m-thick pelagic and hemipelagic carbonated sedimentary sequence overlying the oceanic igneous crust, containing up to 80 wt % CaCO_3_ and 2.5 wt % organic carbon (see figs. S2 and S3). This sequence ranks among the most carbon-rich subducting sediments found at active convergent margins (*15*) (Fig. 2, A and B). Their composition reflects the preferential accumulation of biogenic material over detrital components, driven by strong coastal upwellings and vigorous primary productivity typical of west-facing equatorial margins (*17*, *21*). The overall depletion of lithogenic elements such as Th, Nb, K_2_O, and TiO_2_ relative to the Bulk Continental Crust (BCC) indicates a strong biogenic dilution and a limited contribution from terrigenous sources (see fig. S4). Because of their elevated carbonate content, bulk PB sediments exhibit exceptionally high CaO/TiO_2_ ratios, matched only by nearby carbonate-rich sediments from Peru and Central America (Fig. 2, A and B).

In addition to their high total carbon content, PB sediments reveal two distinctive geochemical features that set them apart from other subducting materials worldwide. First, elevated surface-water productivity enhances microbial respiration of sinking organic matter, promoting the precipitation of copious amounts of biogenic barite (*26*). The continued accumulation of this barite during sedimentation leads to exceptionally high Ba concentrations in the bulk sediment, with values up to an order of magnitude greater than those of continental-derived sediments. This results in elevated Ba/K_2_O ratios, a well-established proxy for high-primary productivity (Fig. 2, B and C). Second, because heavy Zn isotopes are preferentially incorporated into CaCO_3_ and PB sediments contain abundant carbonate (*27*, *28*), these sediments exhibit notably heavy δ^66^Zn values, reaching up to 0.61 ± 0.02‰ (Fig. 2, C and D, expressed as the per mil deviation of measured ^66^Zn/^64^Zn ratios relative to the JMC-Lyon standard; see data S1 and fig. S3). Although some PB sediment samples have carbonate contents and CaO/TiO_2_ ratios similar to those from comparable latitudes in the Western Equatorial Pacific, they exhibit distinctly heavier δ^66^Zn values (*29*). Western Equatorial Pacific sediments primarily reflect the average lithogenic δ^66^Zn composition [0.27 ± 0.07‰; (*30*)] (Fig. 2D). This isotopic offset likely signals the influence of carbonate precipitation in a region of high primary productivity, where lighter Zn isotopes are preferentially incorporated into organic matter, enriching the seawater pool (and the carbonated precipitated from it) in heavier Zn isotopes (*28*, *31*). In contrast, the lower surface-water productivity of the Western Equatorial Pacific (*17*) aligns with its more continental-like Ba/K_2_O ratios (*32*) (Fig. 2C).

Numerous studies have shown that the strong biogeochemical signature of the Eastern Equatorial Pacific sediments is reflected in the compositions of arc-front volcanoes, not only in Colombia (*21*, *33*) but also throughout the entire Eastern Equatorial Pacific margin (*16*, *34*). For example, high-Mg# (~60) andesites and basalts from the Colombian and Central American arc-front display remarkably high Ba contents and elevated Ba/K_2_O ratios, resulting from the subduction of Ba-rich sediments from this region. Furthermore, the subduction of substantial amounts of CaCO_3_ has been identified as a major contributor to the large volumes of CO_2_ [~1.3 million tonnes (Mt) C/year] released by fumarolic gases from North Andean arc-front volcanoes (*35*).

### The Magdalena Volcanic Field

The MVF is built on an ~60-km-thick continental crust and overlies the late Paleozoic to early Mesozoic metamorphic and igneous complexes of Colombia’s Central and Eastern Cordilleras (*36*, *37*). The MVF consists of ~30 scoria cones, pyroclastic tuff rings, and associated lava flows with compositions ranging from silica-undersaturated nephelinites-basanites to alkaline basalt and subalkaline andesites (*22*, *38*). Given their stratigraphic position, geomorphology, and preservation, it has been suggested that the MVF was emplaced during the Quaternary, with possible historical volcanic activity (*22*). Here, we focus exclusively on the basanitic and nephelinitic lavas from the MVF, which we will refer collectively as the Magdalena basanite-nephelinite (MBN) suite.

**Fig. 3.**
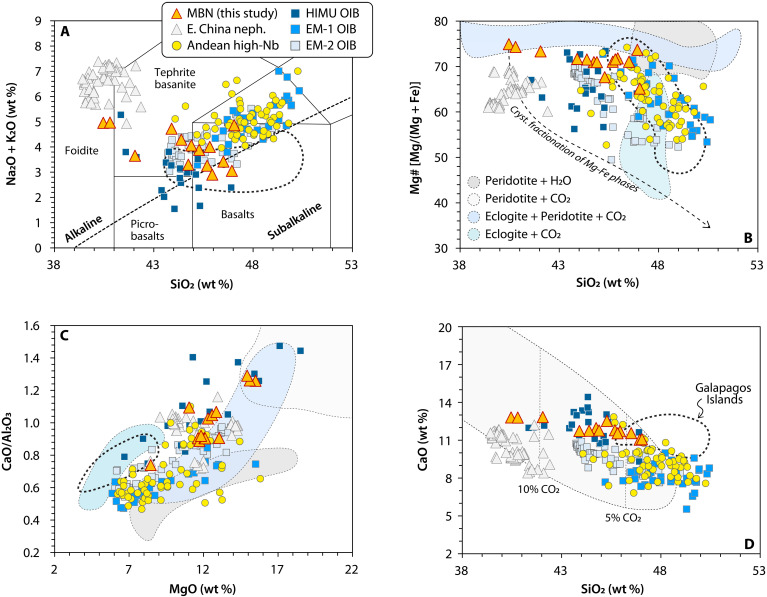
Major elements systematics from MBN suite rocks, Andean high-Nb basalts, Eastern China nephelinites, and multiple OIB type rocks (HIMU, EM-1, EM-2, and Galapagos). Compiled data obtained from the Geochemistry of Rocks of the Oceans and Continents (GEOROC) database, high-Nb global sodic basalt compilation from ref. (*40*), and HIMU basalts (including Zn isotope data) from ref. (*10*). Data were filtered to include only fresh lavas [loss on ignition (LOI) < 3 wt %] with high MgO contents (>6 wt %). (**A**) SiO_2_ versus Na_2_O + K_2_O showing the silica-undersaturated character of MBN rocks and its similarities with Eastern China nephelinites and HIMU-type lavas. (**B** and **C**) SiO_2_ plotted against Mg# and MgO plotted against CaO/Al_2_O_3_ showcasing experimental melt data of carbonated peridotite, carbonated eclogites, hydrous peridotite, and reactive carbonated eclogite melts with peridotite (*57*, *58*, *102*, *103*). (**D**) SiO_2_ plotted against CaO showing the field of experimentally determined primary CO_2_ concentrations (*14*) and the potentially high primary CO_2_ contents of MBN rocks.

**Fig. 4.**
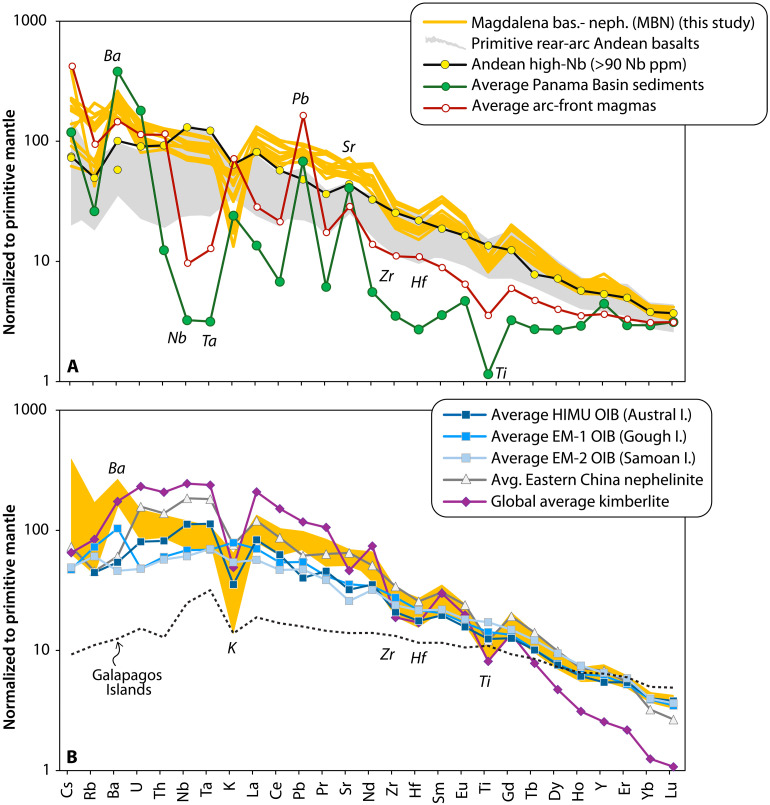
Primitive mantle normalized trace element concentration of MBN rocks. (**A**) Andean high-Nb basalts and average highest-Nb Andean rear-arc rocks show no systematic appearance of negative Ti or Zr-Hf anomalies that are strongly displayed by MBN lavas. Also shown in (A) are the average composition of arc-front rocks from the Colombian Andes (*16*) and the average composition of carbonate-rich PB sediments (*21*). PB sediments differentiate from continental subducting materials by having notably more Ba and Sr but an overall depletion in REEs and alkali elements. (**B**) MBN and the same rock suites as presented in Fig. 3 are also shown for comparison. MBN rocks show much more resemblance to Eastern China nephelinites and average kimberlitic melts (*39*). Primitive mantle composition from ref. (*104*).

MBN rocks display a major element composition that extends from slightly alkaline basalts to basanites and foidites/nephelinites (Fig. 3A and fig. S5; major and trace element concentrations can be found in data S2). These rocks display high Mg# (~70), but their magmatic evolution follows a gentle slope in the SiO_2_-Mg# space, which contrasts to the steeper decreasing trends typically attributed to crystal fractionation of ferromagnesian phases (Fig. 3B). Their trace element patterns show strong enrichment in rare earth elements (REEs) and exhibit no Nb-Ta or Pb anomalies, setting them apart from typical arc-front magmas of the Northern Colombian Andes (Fig. 4A). To contextualize the primitive major and trace element composition of the MBN rocks, we compare them with other Andean intraplate rocks, OIB end-members, and carbonated peridotite melts (e.g., Eastern China nephelinites). At first glance, MBN rocks show no major or trace element affinities with other primitive, high-Nb, rear-arc lavas from the entire Andean Cordillera, all of which display lower CaO concentrations and CaO/Al_2_O_3_ ratios (Fig. 3, C and D), lower trace element abundances, and the absence of a pronounced K, Zr-Hf, and Ti negative anomalies (Fig. 4A and fig. S6 for an extended comparison to other intraplate-like convergent margin lavas). Clear geochemical differences also exist between MBN rocks, enriched mantle type 1 (EM-1) and enriched mantle type 2 (EM-2) lavas, and the nearby Galapagos OIBs, all of which have higher K and Ti and flatter trace element patterns (Fig. 4B). Instead, MBN rocks more closely resemble HIMU-type OIBs and intraplate sodic nephelinites from Eastern China. These suites share the high CaO/Al_2_O_3_ ratios, elevated CaO contents, silica-undersaturated character, and analogous K, Zr-Hf, and Ti negative anomalies (Figs. 3 and 4B). Despite having steeper REE patterns, global average kimberlitic melts (*39*) also display comparable K, Zr-Hf, and Ti negative anomalies to those found in MBN lavas (Fig. 4B). However, one singular aspect that differentiates MBN rocks from other volcanic suites is their pronounced positive Ba anomaly (Fig. 4). This appears to be a distinctive feature of MBN on a global scale, as shown by a comparison with a global compilation of Quaternary high-Mg, primitive, sodic basalts (see fig. S6).

**Fig. 5.**
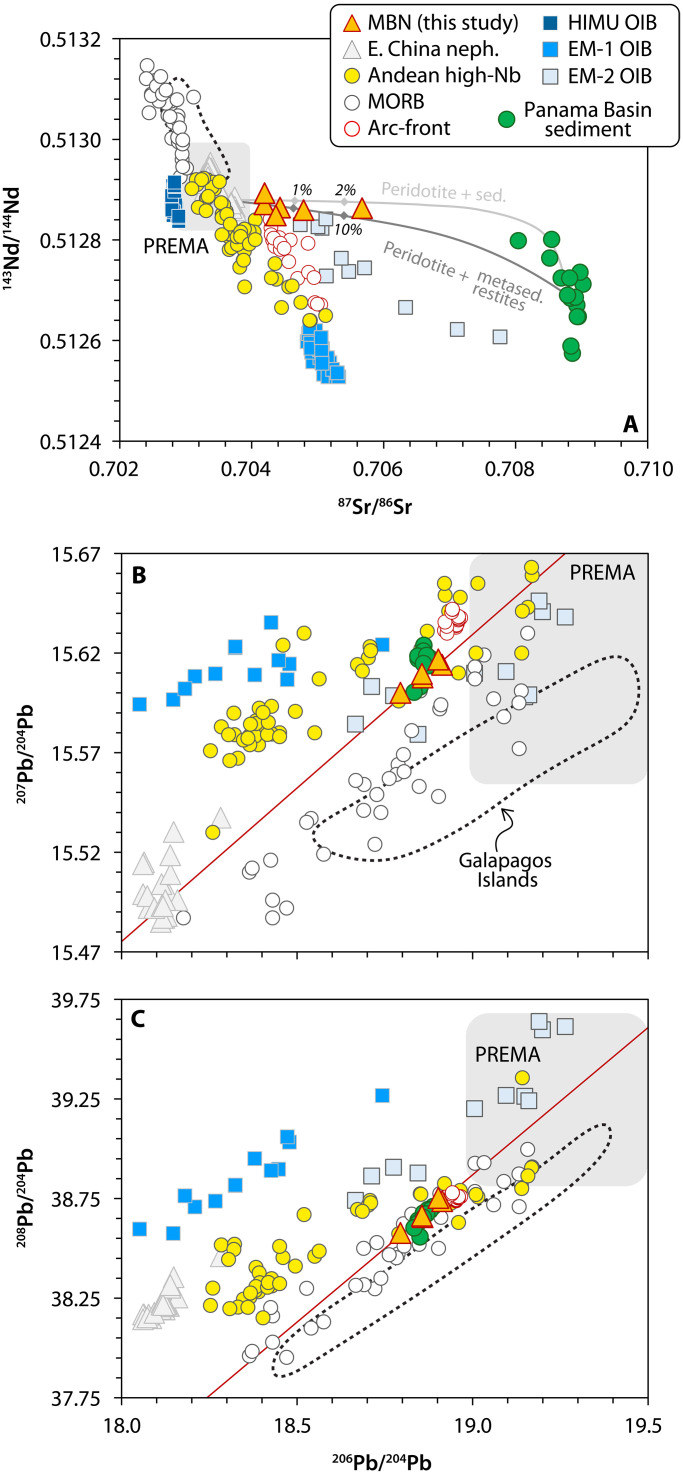
Radiogenic Sr, Nd, and Pb isotopic compositions of MBN rocks. Same rock suites as presented in Fig. 3 are also shown for comparison. (**A**) ^87^Sr/^86^Sr plotted against ^143^Nd/^144^Nd displaying the decoupling of MBN rocks from the mantle array imposed by Andean high-Nb basalts, Eastern China nephelinites, Galapagos Islands, and the Eastern Pacific Rise (EPR) MORBs. (**B** and **C**) ^206^Pb/^204^Pb plotted against ^207^Pb/^204^Pb and ^208^Pb/^204^Pb showcasing the overlapping of MBN rocks with PB sediments and their shift away from the PREMA field (*40*).

MBN rocks exhibit ^143^Nd/^144^Nd ratios similar to a Prevalent Mantle (PREMA) or HIMU–like mantle source (*40*), but their more radiogenic ^87^Sr/^86^Sr ratios shift their isotopic compositions toward that of PB sediments (Fig. 5A), partially overlapping with EM-2 lavas. Their Pb isotopic ratios are relatively homogeneous and fall within the field of PB sediments, plotting slightly outside the PREMA domain (Fig. 5, B and C). To some extent, MBN coincides with an EM-2 mantle composition in the ^206^Pb/^204^Pb versus ^207^Pb/^204^Pb space; however, they follow contrasting linear arrays in terms of ^208^Pb/^204^Pb versus ^207^Pb/^204^Pb isotopes. MBN rocks plot near other Andean high-Nb basalts, at slightly less radiogenic Pb isotopic composition than arc-front lavas (Fig. 5, B and C). The δ^66^Zn isotopic compositions of MBN rocks (δ^66^Zn = 0.32 ± 0.01 to 0.37 ± 0.02‰) are remarkably similar to those found in HIMU basalts and some Eastern China nephelinites (*12*) but are heavier than primitive mid-ocean ridge basalts (MORBs; δ^66^Zn = 0.22 ± 0.06‰) (*41*) and the average peridotitic mantle (δ^66^Zn = 0.16 ± 0.06‰) (*42*) (see fig. S7; whole-rock radiogenic and stable isotopic compositions can be found in data S2).

### Geochemical evidence of carbonated-silicate melts

**Fig. 6.**
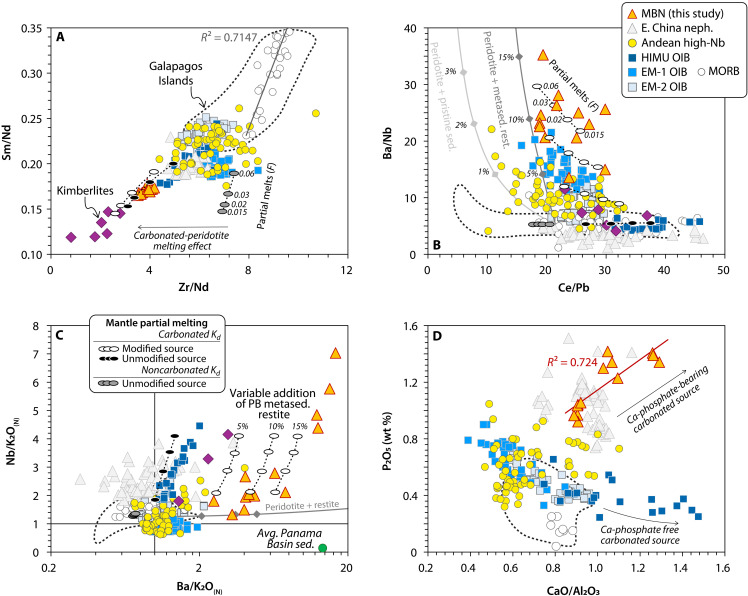
Geochemical systematics of carbonate and noncarbonate related mantle melts. (**A**) Zr/Nd versus Sm/Nd ratios displaying the decoupling from MBN rocks, Eastern China nephelinites, and kimberlitic melts from noncarbonated mantle melts (i.e., MORBs, EM-1, EM-2, and Galapagos Islands) due to the impact of carbonated mantle lithologies in controlling the partitioning of HFSEs (*48*). (**B** and **C**) Ce/Pb plotted against Ba/Nb, and Ba/K_2_O_(N)_ plotted against Nb/K_2_O_(N)_ showcasing the strong fractionation of MBN rocks when compared to other carbonate-related mantle melts. Elevated Ba/Nb and Ba/K_2_O_(N)_ ratios require the participation of a Ba-rich component likely represented by barite-rich PB subducted sediments. Nb/K_2_O_(N)_ and Ba/K_2_O_(N)_ normalized to primitive mantle values from ref. (*104*). Vertical and horizontal black lines in (C) intersect at 1. Partial melting models describe the evolution of partial melts from a modified (with variable proportion of PB metasedimentary restites) and unmodified mantle source, using partition coefficients (*K*_*d*_) in equilibrium with a carbonated peridotite (*48*). Unmodified mantle melts computed using *K*_*d*_ in equilibrium with noncarbonated lithologies are also shown for comparison. For further details, see text S1. A complete array of modeled trace elements can be found in fig. S10. (**D**) CaO/Al_2_O_3_ plotted against P_2_O_5_. The best fit for P_2_O_5_ concentrations corresponds to the melting of a P-rich mantle (*69*) modified by carbonate and apatite-bearing metasedimentary restites.

The compositional similarity of MBN rocks with intraplate nephelinites, HIMU lavas, and kimberlitic melts, each potentially derived from mantle sources containing variable proportions of carbonated components (*10*, *12*, *39*, *43*), initially suggests a comparable petrogenesis for the North Andean rear-arc region. A carbonated mantle source for MBN rocks is further supported by their lower Zr/Nd (Fig. 6A) and elevated Eu/TiO_2_ ratios (fig. S8), a common feature observed not only in kimberlites and intraplate nephelinites but also in carbonatites (*44*) and mantle xenoliths metasomatized by carbonatitic melts (*45*–*47*). This stems from the higher mineral-melt partition coefficients of Zr, Hf, and TiO_2_ in carbonated-peridotite systems, a consequence of the low solubility of high-field-strength elements (HFSEs) in carbonated-silicate melts (*48*). Low-degree partial melting models (*F* = 0.015 to 0.06) of a carbonated peridotite mantle can reproduce the Zr/Nd and Eu/TiO_2_ ratios of MBN rocks, intraplate nephelinites and kimberlites (Fig. 6A and fig. S8) (see text S1 for details on the geochemical models). In contrast, melts derived from carbonate-poor mantle sources (i.e., MORBs, EM-1, EM-2, or Galapagos OIBs with lower CaO content and low CaO/Al_2_O_3_ ratios; see Fig. 3, C and D), are better explained by carbonate-free element partitioning data (Fig. 6A).

Although a carbonate component appears essential to account for the composition of MBN lavas, it alone cannot explain the elevated Ba/Nb and Ba/K_2_O ratios compared to other carbonated mantle melts (Fig. 6, B and C). The broadly similar partitioning behavior of Ba and Nb (D_Ba_ ≈ D_Nb_) in typical mantle minerals suggests that Ba/Nb variations should mainly reflect source heterogeneities (*49*). This is exemplified by the elevated Ba/Nb ratios in EM-1 lavas, which have been associated with the incorporation of ancient crustal materials in their mantle source (*50*). The relatively low ^143^Nd/^144^Nd and high Ba/Nb ratios of rear-arc, Andean high-Nb basalts (Figs. 5A and 6B), have led to the proposal that an EM-1-type mantle dominates the background mantle beneath the Andean subduction zone (*51*). However, linking the high Ba/Nb in MBN lavas to an EM-1 mantle source is problematic. MBN rocks exhibit even higher Ba/Nb ratios and lack other geochemical indicators of continental inputs, such as correlations with Ce/Pb or unradiogenic Nd isotopes (see fig. S9). Moreover, their negative K anomalies, high Mg#, and low SiO_2_ contents make the involvement of an evolved and enriched continental end-member unlikely.

The origin of negative K anomalies in deeply sourced intraplate magmas has been attributed to the presence of carbonate in the mantle, which can promote the stabilization of K-rich residual phases such as phengite and phlogopite at low to intermediate pressures (3 to 9 GPa) or liebermannite (KAlSi_3_O_8_) at higher pressures (9 to 22 GPa) (*7*, *18*, *52*, *53*). However, despite the pronounced K depletion observed in intraplate nephelinites, HIMU basalts, and kimberlites (Fig. 4B), none of these lavas exhibit elevated Ba/K_2_O ratios, indicating a similar partitioning behavior of Ba and K_2_O during partial melting of a carbonated peridotite containing a K-rich phase. For example, batch melting models of carbonated-silicate melts that incorporate residual phlogopite adequately reproduce the elevated Nb/K_2_O_(N)_ that characterizes intraplate nephelinites and HIMU lavas but are unable to replicate the high Ba/K_2_O ratios of MBN rocks at equivalent Nb/K_2_O_(N)_ values (Fig. 6C). This implies that the MBN lavas require an additional Ba-rich component in their source region, one capable of overriding the geochemical signature imparted by residual phlogopite or other K-rich phase alone. These compositional features are absent in other primitive Andean high-Nb basalts, indicating that the Ba-rich nature of the MBN mantle source is a region-specific characteristic, likely arising from the particular geological conditions of the northernmost Andean chain. On this basis, the peculiar composition of subducted PB sediments may represent not only the carbonated component required to transform the mantle beneath the MBN but also the source of their distinctively elevated Ba/K_2_O and Ba/Nb ratios. The unusually high Ba content in these sediments, caused by biogenic barite precipitation under high-productivity conditions, makes them strong candidates to explain the Ba enrichment in MBN magmas. This, in turn, suggests that the mantle source of the MBN suite is closely linked to recent marine biogeochemical cycles and the deep-sea sedimentary history of the PB.

### Mantle modification driven by carbonate-rich PB sediments

The primitive nature of the MBN, evidenced in their high Mg# values (up to 75), indicates that they cannot be formed by direct melting of carbonated PB sediments, as has been proposed for other high-carbonate flux margins (*54*). Because experimental partial melts of carbonated MORB-eclogite, analogs of carbonate incorporation into oceanic crust under high-pressure conditions (*55*, *56*), are characterized by low MgO and high SiO_2_ contents, they are also unsuitable as primary sources for MBN lavas (Fig. 3, B and C). This suggests that the MBN suite did not derive directly from a carbonated slab or pure sediment melt. In contrast, both experimental melts of carbonated peridotite (*14*, *57*), and melts produced by melt-rock reactions between carbonated MORB-eclogite melts and peridotite (*58*) show a closer resemblance to MBN rocks (Fig. 3, B and C). Nonetheless, the isotopic composition of the MBN does not support the involvement of a MORB-like mantle source. Instead, the enriched Sr isotopic signature is more consistent with a mixing scenario between a PREMA-like mantle and PB sediment (Fig. 5A). Yet, because the Sr concentration in bulk PB sediments far exceeds that of a mantle peridotite, 1 to 2% addition of pristine carbonated sediment would shift the isotopic composition of the mixture toward more radiogenic ^87^Sr/^86^Sr ratios than those observed in MBN rocks. Aside from Ba and Sr, PB sediments are relatively depleted in trace elements compared to continental materials, but they are still more enriched in Pb relative to the mantle (Fig. 4A). Therefore, mixing small amounts (1 to 2%) of pristine sediment into a peridotite mantle would also imprint low Ce/Pb ratios (Fig. 6B). The MORB-like Ce/Pb ratios (>20) of MBN lavas thus preclude the direct addition of pristine PB sediments in their mantle source.

Mass balance calculations of carbonate inputs, combined with the high CO_2_ emissions from arc-front fumaroles (~1.3 Mt C/year), suggest that at least one-third of the subducted carbonate in Colombia is recycled at the arc-front (*35*). Furthermore, the widespread geochemical signatures of subducted sediments preserved in Colombian and global arc-front magmas suggest that these materials rarely remain compositionally pristine during their passage through the subduction channel (*21*, *59*, *60*). In addition to undergoing partial melting beneath the arc-front, subducted sediments may also undergo dehydration and decarbonation reactions during the early stages of subduction, releasing fluid-mobile elements and CO_2_ into the mantle wedge (*61*). As a result, extensively modified sedimentary lithologies that reach the rear-arc are expected to be depleted in elements preferentially extracted by fluids and melts, such as Pb and Sr. These residues left after partial melting, hereafter refer to as “metasedimentary restites,” are also thought to be denser than the overlying mantle wedge and may sink to greater depths along the slab-mantle interface (*62*). As proposed for the origin of mantle heterogeneities in OIBs and back-arc basin basalts, these deeply buried restites may ultimately act as key enrichment agents in the deepest regions of the mantle (*63*).

To model the composition of deeply buried, subducted metasedimentary restites, we conducted inverted batch melting calculations using a bulk carbonated PB sediment in equilibrium with clinopyroxene + carbonate + garnet + apatite, a mineralogical assemblage consistent with experimental melts of carbonate-rich sediment at sub-arc depths (*33*, *64*, *65*) (see text S1 for details). We predict that these restitic carbonate-bearing lithologies lead to overprinting of the ambient peridotite, generating a modified mantle region that serves as the source of the MBN lavas. This chemically and lithologically heterogeneous mantle might be similar, although not identical to MARID-veined (mica-amphibole-rutile-ilmenite-diopside) or PIC-veined (phlogopite-ilmenite-clinopyroxene) mantle assemblages, which are often linked to carbonate metasomatism of highly alkaline and kimberlitic magmas (*66*). Figure 6 (A to C) and fig. S10 show that low-degree partial melts (*F* = 0.015 to 0.06) in equilibrium with a phlogopite/garnet-bearing lherzolite reproduce well the composition of MBN magmas, including their elevated Ba contents and high Ba/Nb(K_2_O) ratios (Fig. 6C). In our model, 5 to 15% addition of PB metasedimentary restites to a peridotite mantle are required to successfully reproduce MBN compositions. A group of samples displays even higher Ba/Nb(K_2_O) ratios (Fig. 6, B and C), stronger K_2_O anomalies, and lower Rb contents (Fig. 4A) but shows no other distinctive elemental or isotopic composition, possibly signaling variable proportions of residual phlogopite in the mantle source underneath the MBN.

It is also noteworthy that MBN rocks, along with the intraplate nephelinites discussed here, are remarkably enriched in P_2_O_5_ (Fig. 6D), a distinctive feature also observed in natural carbonatitic melts (*44*, *67*). Despite the high incompatibility of phosphorous (P) in common mantle minerals in equilibrium with both silicate and carbonatitic melts, achieving such elevated P_2_O_5_ concentrations from a lherzolitic mantle source seems unlikely, even at extremely low melting degrees (**F* =* 0.01) (*68*, *69*). Hence, mantle-derived melts with high P_2_O_5_ content (>~0.7 wt %) likely require a mantle source containing both carbonate and apatite (*69*). The bulk P_2_O_5_ composition of PB sediments is, within error, similar to global subducting sediment (GLOSS) (~0.14 wt %); however, the sedimentary column contains apatite-rich layers associated with accumulated fish debris (*33*). The formation of apatite-rich metasedimentary restites can thus be linked to their original modal composition. More plausibly, residual apatite may form as a reaction product during the partial melting of subducted sediment (*70*). In this scenario, apatite stability may be enhanced by the carbonate-rich composition of PB sediments, arguably because its solubility is inversely correlated with the CaO content of the system (*71*). This interpretation is consistent with the common occurrence of apatite in carbonatites (*72*) and the frequent association of apatite-bearing mantle xenoliths with carbonate metasomatism (*46*, *47*). Therefore, we propose that the elevated P_2_O_5_ content of MBN lavas reflects melting of a mantle modified by carbonate- and apatite-bearing metasedimentary restites. The high P_2_O_5_ content in MBN lavas further indicates that any apatite initially present in their mantle source was likely fully consumed during mantle melting due to the high solubility of apatite in basaltic systems (*73*). The compositional deviation of MBN rocks and Eastern China nephelinites from MORBs, Galapagos, EM-1, and EM-2 OIBs in terms of P_2_O_5_ and CaO/Al_2_O_3_ underscores the influence of carbonate- and apatite-rich lithologies in their carbonated mantle sources (Fig. 6D). In addition, strong correlations between P_2_O_5_ and other carbonate-related geochemical proxies, such as Zr/Nd and Eu/TiO_2_ are observed across our dataset (see fig. S8).

The influence of recent subduction inputs on the MBN mantle source is further confirmed by the nearly identical Pb isotopic compositions of MBN rocks and present-day PB sediments (Fig. 5, B and C). This implies that, despite being strongly processed through the subducting channel, subducted metasedimentary restites can still dominate the Pb isotopic budget relative to the ambient mantle peridotite (see fig. S11). However, the Nd-Hf isotopic systematics of MBN lavas deviate markedly from the expected mixing trends between PREMA and PB sediments, indicating that not all trace element and isotopic signatures are controlled by subducting components (see fig. S12). This is particularly true for Hf, given its extremely low abundance in pristine PB sediments (see Fig. 4A). Furthermore, because Hf behaves incompatibly during sediment melting, metasedimentary restites are expected to display even lower Hf concentrations than mantle lithologies (see fig. S11). This likely explains why the Nd-Hf isotope signatures of MBN lavas plot closer to the mantle array than in the Sr-Nd or Pb-Pb isotopic spaces.

### Zn isotopic evidence for carbonated metasedimentary restites

To more precisely assess the carbonate contribution to MBN magmas, we combine Zn isotopic data with other elemental and isotopic tracers that signal the presence of PB carbonates in their mantle source. Recent findings have indicated that HIMU-type OIBs and Eastern China nephelinites display heavy Zn isotopic compositions that cannot be fully explained by partial melting of a peridotite mantle source (*10*, *11*). Intermineral Zn fractionation during mantle melting produces a melt-mantle isotopic offset (Δ^66^Zn_Melt-Mantle_) between ~0.06 and 0.1‰ (*42*, *74*). Accordingly, partial melting of a peridotite source having δ^66^Zn ≈ 0.16‰ would produce Zn isotopic compositions that are too light to explain the δ^66^Zn values observed in MBN rocks. Although partial melting of some sublithospheric mantle lherzolites [δ^66^Zn = up to 0.27‰; (*75*)] could potentially replicate the heavy Zn isotopic compositions of some mantle-derived melts, this process alone cannot fully account for the geochemical systematics observed in MBN. Therefore, the involvement of a carbonated component with isotopically heavy Zn is required.

**Fig. 7.**
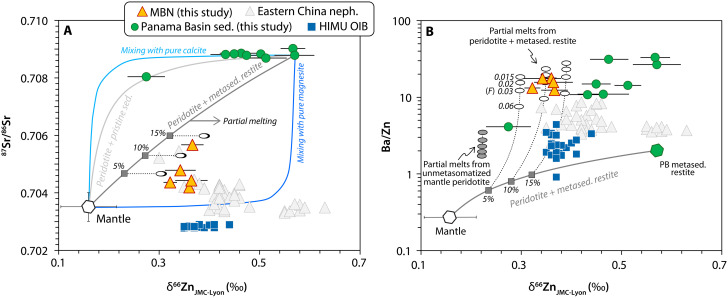
Zn isotopic systematics of MBN rocks. Other well-characterized deeply sourced carbonated mantle melts (i.e., HIMU basalts and Eastern China nephelinites) are also shown for comparison. The Zn isotopic compositions of MBN are explained by mixing (gray line) and further melting (white ovals) of a modified mantle wedge with a metasedimentary restitic component. (**A**) δ^66^Zn versus ^87^Sr/^86^Sr. The compositions of calcite and magnesite, often invoked as potential recycled carbonates, are from ref. (*11*). Mantle composition in terms of Sr isotopes is taken as PREMA (*40*), whereas its Zn composition is taken as the average value of mantle peridotites (*42*). (**B**) δ^66^Zn versus Ba/Zn. Partial melts of an unmodified peridotite mantle (gray ovals) are shown for comparison. They display notably lower Zn isotopic composition and Ba/Zn ratios than MBN rocks. Further details of the modeling can be found in text S2.

Our Zn isotopic data reinforce the notion that simple mixing trajectories between pristine PB sediment and a peridotite mantle do not reproduce the composition of MBN rocks (Fig. 7A). This arises from the high Sr and low Zn concentrations of oceanic carbonates, further indicating that prior elemental transformation is required for these materials to serve as adequate mixing end-members. The mixing trend between pristine PB sediments and mantle peridotite also mirrors the incorporation of pure calcite because PB deep-sea carbonates are predominantly composed of CaCO_3_ (up to 60 wt %). Given that the mixing path between peridotite and calcite strongly deviates from the compositions of other carbonated peridotite melts (e.g., HIMU basalts and Eastern China nephelinites), pure Mg(Ca)-carbonates such as magnesite and dolomite, which are characterized by higher Zn and lower Sr concentrations, have been considered more viable carbonated contributors in the mantle (*29*, *76*). However, it is important to note that these types of carbonates are rarely formed during seafloor sedimentation and are thus nearly absent in near-trench sediments (*13*). This has led to the suggestion that magnesite or dolomite may instead form either through high-pressure transformation of calcite (*11*) or as by-products of olivine carbonation reactions (*77*). Although such transformations are feasible under high pressure-temperature (*P*-*T*) mantle conditions (*3*, *53*), and first-principles calculations indicate a preference of Zn for stiffer bonding environments such as in MgCO_3_ (*78*), no experimental studies or natural case examples have yet demonstrated whether Mg-carbonates retain the heavy Zn isotopic composition of their biogenic calcite precursors. Primary Mg-rich calcites from carbonated peridotite xenoliths have Zn and Sr contents in similar proportions to those found in ocean sediments (*79*). Yet, they lack many of the alkali elements and P that are essential for the petrogenesis of MBN rocks. Moreover, it remains unclear if the sole presence of Mg-carbonates can facilitate the stabilization of Ca-phosphate (i.e., apatite) necessary to account for the elevated P_2_O_5_ contents in the MBN mantle source. Last, an inherent limitation of pure carbonate recycling in the mantle, especially for phases such as calcite, is their positive buoyancy relative to the surrounding peridotite, which inhibits its transport to greater depths.

Eolian and riverine inputs, combined with in situ precipitation within the sediment column, strongly influence seafloor sedimentation, resulting in deep-sea carbonates near trenches containing variable proportions of detrital and authigenic phases (*80*, *81*). As a consequence, pure carbonate sedimentary sequences are virtually absent near active convergent margins. Instead, this complex sedimentary makeup leads to an elemental enrichment in major oxides (i.e., TiO_2_, Al_2_O_3_, and MgO), alkali (i.e., K, Rb, and Cs), HFSEs (i.e., Ti, Nb, and Zr), U, Th, and REEs, all of which can influence the stability of mineral assemblages during prograde metamorphism and/or melting along the subduction channel. Experimental residues from the melting of natural carbonated sediments equilibrate not only pure carbonate phases but also variable amounts of pyroxene, garnet, phlogopite, liebermannite, apatite, and other accessory phases (*64*, *65*, *81*, *82*). Compared to pure Mg(Ca) carbonate phases, these so-called “carbonated metasedimentary restites” are not only more efficient carriers of major and trace elements into the deep Earth, but they are also denser than the surrounding mantle and therefore negatively buoyant, allowing them to sink readily to greater depths. This perspective offers a more viable alternative to idealized models that consider pure carbonate phases as the dominant carbonate contributors to the mantle sources of carbonated peridotite melts.

Using the same proportions outlined in Fig. 6, our proposed model aligns well with the geochemical systematics that arise from mixing the peridotite mantle with PB metasedimentary restites in terms of δ^66^Zn, ^87^Sr/^86^Sr, and Ba/Zn ratios (Fig. 7). Following partial melting in equilibrium with varying proportions of pyroxene and garnet, these restites become progressively enriched in Zn relative to Sr. This shift generates mixing trajectories that lie between the pure calcite and pure magnesite end-members, closely intersecting the compositional field of MBN rocks. For the same reason, PB metasedimentary restites also have lower Ba/Zn ratios than their pristine precursors. However, because Zn is less incompatible than Ba in mantle mineral assemblages, and given the minimal Zn isotopic fractionation during partial melting, low-degree melts derived from a modified mantle source can successfully reproduce both the high Ba/Zn ratios and the heavy Zn isotopic compositions of MBN rocks.

### Implications for carbonate recycling into the deep mantle

**Fig. 8.**
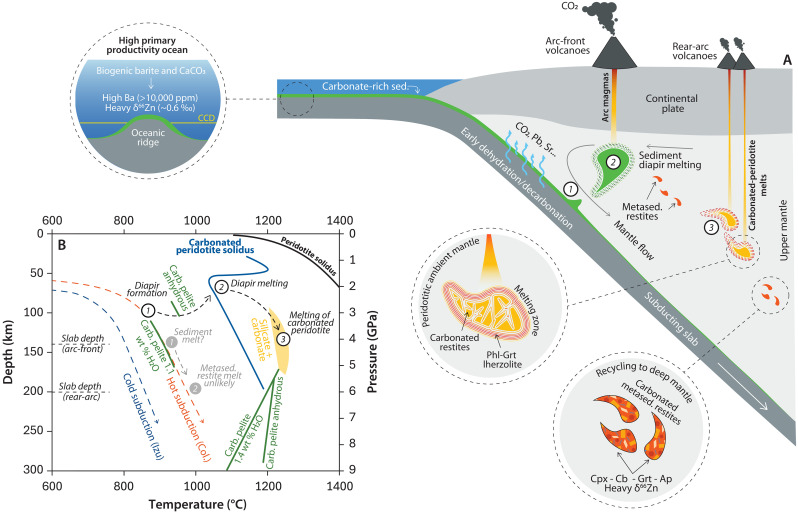
Proposed formation of the metasedimentary restites as deep mantle carbonate contributors and the genesis of the MBN. (**A**) Earth’s surface conditions determine the elemental and isotopic composition of carbonate-rich subducting sediments. Once subducted, these sediments undergo dehydration and decarbonation reactions and can later form buoyant diapirs that rise into the mantle wedge beneath arc-front volcanoes. Following partial melting, metasedimentary restites are transported deeper along the slab-mantle interface, contributing to the chemical heterogeneity in the source region of carbonated peridotitic melts. This process transfers carbon, incompatible elements, and a heavier Zn isotopic signature to the convecting mantle. (**B**) *P*-*T* path of the Colombian subduction zone (*84*) and its relationship with the solidus of carbonated pelites under subduction zone conditions (*18*, *53*, *64*, *105*). Melting at the slab-top interface at sub-arc and rear-arc depths presents substantial thermal challenges, especially given the high-temperature solidus of dry carbonated pelites. Instead, we favor a model in which metasedimentary restites are transported into the mantle wedge, where they cross the carbonated peridotite solidus and may enter the stability field of carbonated-silicate melts (*53*). Cpx, clinopyroxene; Cb, carbonate; Grt, garnet; Ap, apatite.

The sedimentary input to the Colombian trench is unevenly distributed along the margin, reflecting the complex bathymetry imposed by the subduction of ridges and extinct rifts within the PB (*21*). These oceanic structures, being shallower than the surrounding seafloor, help maintain sediment accumulation above the local carbonate compensation depth (CCD), thus promoting more efficient transfer of carbonate-rich sediments into the mantle at convergent margins (*21*) (Fig. 8A). The subduction of the Carnegie Ridge and Malpelo Rift beneath the MBN region thus suggests a preferential delivery of carbonate-rich sediments to its underlying mantle source. The seismically imaged thick sediment pile near the trench [600 to 1000 m; (*83*)], combined with the composition of the incoming sediment column and the hot thermal regime of the Colombian subduction zone (*84*), provides favorable conditions for the development of buoyant sediment diapirs rising through the sub-arc mantle (*62*). This mechanism has been previously proposed for Colombia (*21*, *33*) as well as in other Cordilleran arc systems (*59*, *85*). Experimental and thermodynamic models (*86*, *87*) indicate that once sediment diapirs enter the mantle wedge, they undergo extensive partial melting (*F* = 0.3 to 0.5), contributing not only to the generation of intermediate arc-front magmas but also in transferring a portion of subducted carbon into the continental crust and Earth’s surface (*4*). Numerical models further predict that in hot subduction zones, such as the one underneath Colombia (*84*), sediment diapir restites may become negatively buoyant relative to the mantle shortly after melt extraction, leading to their rapid entrainment into deeper sections of the subduction channel (*62*) (Fig. 8, A and B). In this scenario, MBN lavas would originate from a hot region of the mantle wedge, rather than directly from the slab-top, under *P*-*T* conditions above the experimentally determined solidus of carbonated peridotite and within the range of carbonated-silicate melts derived from carbonated pelites at mantle depths (Fig. 8B).

Alternatively, the MBN may be an exceptional case. Recent seismic imaging has identified the development of a slab gap or tear at ~200 km depth beneath the region (*24*), potentially creating anomalously hot mantle conditions in the absence of subducting lithosphere or inducing decompression melting via asthenospheric upwelling (*38**,* *88*). In these instances, asthenospheric mantle flow could raise slab-edge temperatures enough to promote the melting of a modified mantle by carbonated metasedimentary restites. This scenario would require a slab tear or gap large enough to increase slab temperatures by as much as ~200°C to cross the carbonated peridotite solidus (Fig. 8B). Although we favor the diapir model based on *P*-*T* constraints and arc-front evidence, MBN compositions are ultimately governed by the nature of biogenic carbonates entering the trench and their transformation into carbonated metasedimentary restites at sub-arc depths. This would be the case across both sediment diapirism and slab-top/edge melting scenarios (Fig. 8B).

Contrary to global carbon flux estimates that suggest limited carbon recycling into the deep mantle (*4*), our findings reveal that, even under warm slab geotherms, and following sediment diapirism in the hot corner of the mantle wedge, subducted carbonate can survive dehydration and partial melting, remaining partially stable beyond sub-arc depths. Evidently, the *P*-*T* conditions of mantle melting below MVF are relatively shallow (<~200 km) compared to the mantle transition zone (~410 to 660 km) and lower mantle regions (>700 km), where primary carbonated-silicate melts associated with Eastern China nephelinites and OIB HIMU-type lavas are believed to originate (*10*, *43*). Nonetheless, in the absence of additional thermal disturbances, carbonated metasedimentary restites can effectively transport incompatible elements and carbon to greater mantle depths (Fig. 8), likely facilitated by metamorphic reactions such as the transformation of calcite into magnesite and phlogopite into liebermannite under increasing pressure (*82*, *89*). The transformation of apatite to tuite [Ca_3_(PO_4_)_2_], which remains stable down to ~700 km depth, suggests that Ca-phosphates in carbonated metasedimentary restites can dominate the P budget throughout the upper mantle and into the mantle transition zone (*69*). The lower P_2_O_5_ contents of deeply sourced HIMU lavas (e.g., St. Helena and Cook-Austral Islands; see Fig. 6D) could probably result from phase instabilities in Ca-phosphates at even greater mantle pressures (*69*). A comprehensive understanding of barite’s behavior in the deep mantle remains limited. However, recent experimental studies have demonstrated that barite can withstand slab-top *P*-*T* paths and even lower mantle pressures (up to 30 GPa) (*90*), partially supporting its occurrence in high-pressure, high-temperature eclogitic xenoliths (*91*). In addition, the Ba isotope signatures of some E-MORBs have also been attributed to the involvement of subducted sediments containing barite (*92*). This suggests that, at convergent margins influenced by high primary productivity such as Colombia, biologically mediated authigenic barite can serve as an important carrier of Ba into the deep Earth.

The large amount of biogenic carbonate subducted along the Colombian Northern Andes is unique among global convergent margins. Nevertheless, the geochemical similarities among MBN lavas and other carbonated peridotite melts suggest that carbonated metasedimentary restites, formed after partial melting of subducted carbonated sediments, may have served as carriers of carbon and other trace elements into the convecting upper mantle elsewhere in Earth’s history. For example, similar Cenozoic alkaline, carbonate-related mantle melts documented in present-day Myanmar and the Balkan Peninsula may reflect the former subduction of carbonate-rich sediments along the Eurasian margin during the closure of the Tethys Ocean (*93*, *94*). Clearly, not all carbonated peridotite melts discussed here exhibit identical chemical compositions, likely because their mantle sources were modified by different metasedimentary restites derived from compositionally distinct sedimentary precursors. Surface Earth processes, such as oceanic primary productivity or deep-sea carbonate preservation, thus play an important role in shaping the nature of subducting carbonates at convergent margins. For the North Andean case, we show that the biogeochemical signatures of Eastern Equatorial Pacific marine carbonates survive subduction and contribute to convecting mantle heterogeneity, as demonstrated by the geochemical association between Ba-rich biogenic PB sediments and the distinctive Ba enrichment and heavy Zn isotopic composition of MBN lavas. Our study reinforces the strong biogeochemical connectivity within the Colombian subduction zone (*16*, *21*, *33*) and establishes a geochemical link between marine ecosystems and deep carbon cycling over millions of years, highlighting that some compositional traits of deeply sourced mantle melts likely have a biological origin, which is itself regulated by the oceanic and climatic conditions that prevailed on Earth’s surface.

## MATERIALS AND METHODS

### Sample selection and preparation

Samples (*n* = 13) were collected from the Huila region in southern Colombia, covering the volcanic clusters of Isnos-San Agustín, Oporapa, and Acevedo (see fig. S1). Notably, nephelinitic and basanitic rocks are absent in the Moscopán cluster, which outcrops west of the sampled areas and lies closer to the arc-front. On the other hand, nephelinitic rocks appear exclusively in the Acevedo locality, situated further east and thus farther from the arc-front. The analyzed samples include volcanic bombs and rock fragments obtained directly from exposed lava flows. They are petrographically characterized by abundant phenocrysts of olivine and tabular microliths of clinopyroxene and plagioclase embedded in a volcanic glassy matrix. Accessory minerals include magnetite-ilmenite and apatite. For bulk-rock geochemical analyses, clean, unaltered chips were selected under a stereoscopic microscope. These chips were then washed with deionized water and ultrasonically cleaned to eliminate dust and impurities.

PB sediments were retrieved by the Deep-Sea Drilling Program (DSDP) at sites 504 and 84, located ~400 km seaward of the Colombian trench (see fig. S2). Detailed descriptions of the lithostratigraphic columns and individual samples are available in refs. (*21*, *33*). Sediments from each DSDP site show a gradual transition from carbonate pelagic deposits at the base to hemipelagic deposits at the top of the sequence (see fig. S3). Correspondingly, carbonate (CaCO_3_) content increases down-core, whereas the terrigenous fraction increases up-core. Zn isotopic analyses (*n* = 7) were performed on powders from previously analyzed samples for major, trace, and Sr, Pb, Nd, and Hf isotopic compositions from ref. (*21*). Samples were selected to represent the broad compositional variability across both drilled cores and spanning variable CaCO_3_ contents (see fig. S3).

### Whole-rock analyses

About 50 mg of bulk-rock volcanic powders was analyzed for major and trace element composition by inductively couple plasma mass spectrometry (ICPMS) using a quadrupole Thermos iCap at the Instituto de Geociencias (IGeo), Universidad Nacional Autónoma de México (UNAM) (data S2: Major and trace elements) following the procedures described in ref. (*95*). Reproducibility and accuracy of major and trace element data at IGeo are given by the average concentrations and SDs of multiple digestions of different rock standards (AGV-2, BHVO-2, BCR-2, JB-2, and internal standard PS-99-25) and have been reported in previous publications (*33*). The average precision during the analytical session of BHVO-2 was better than 4% (RSD) for most trace elements except for Pb, Cs, Nb, and Cu, for which it remained below 12% (RSD).

About 200 mg of clean handpicked rock chips from a subset of six samples was selected to carry out radiogenic isotopic analyses (data S2: Isotopic ratios). Rock chips were first leached in 6 mol L^−1^ HCl and ultrasonically washed two times before digestion. Whole-rock Sr, Nd, Pb, and Hf isotopic ratios were determined using a Neptune Plus multicollector (MC)–ICPMS at the IGeo, UNAM following the procedures described in refs. (*95*, *96*). The measured ^87^Sr/^86^Sr ratios were corrected for mass bias to an ^86^Sr/^88^Sr = 0.1194 and adjusted to an NIST SRM 987 standard ratio of ^87^Sr/^86^Sr = 0.710230. The average measured SRM 987 was ^87^Sr/^86^Sr = 0.710296 ± 0.000017 (2σ, *n* = 5). The measured ^143^Nd/^144^Nd ratios were corrected for mass bias to ^146^Nd/^144^Nd = 0.72190 and adjusted to a JNdi standard ^143^Nd/^144^Nd = 0.512115 (*97*). During the course of this study, the average measured values for JNdi was ^143^Nd/^144^Nd = 0.512090 ± 0.000008 (2σ, *n* = 7). The Pb isotopic compositions were corrected for mass bias by spiking all samples with SRM 997 Tl solution with a reference ^205^Tl/^203^Tl = 2.3871 and adjusted to the NIST SRM-981 standard values of ^206^Pb/^204^Pb = 16.9356, ^207^Pb/^204^Pb = 15.4891, and ^208^Pb/^204^Pb = 36.7006 (*98*). The average measured Pb isotopic compositions of the NIST SRM-981 was ^206^Pb/^204^Pb = 16.9308, ^207^Pb/^204^Pb = 15.4850, and ^208^Pb/^204^Pb = 36.6795 (2σ = 0.001, 0.005, and 0.0013, respectively, *n* = 6). The measured ^176^Hf/^177^Hf ratios were corrected for mass bias using ^179^Hf/^177^Hf = 0.7325 and further adjusted to ^176^Hf/^177^Hf = 0.282160 of the Hf-Spex standard, which has been intercalibrated and considered identical to the JMC-475 standard (*96*). The average value obtained for Hf-Spex was ^176^Hf/^177^Hf = 0.282150 ± 0.000003 (2σ, *n* = 11). Reproducibility and accuracy of these isotopic measurements were verified using US Geological Survey (USGS) standards BCR-2, BHVO-2, and AGV-2, which were prepared and measured using the same procedures as the rock samples.

About 10 to 20 mg of rock and sediment powders was selected to carry out stable Zn isotopic analyzes. Chemical purifications were conducted following the procedures from ref. (*99*). Samples were loaded in 1.5 mol L^−1^ HBr into 500 μl of PTFE columns filled with AG1-X8 (200 to 400 mesh) anion-exchange resin. Matrix elements were removed by adding an additional 5 ml of 1.5 mol L^−1^ HBr. Zinc was then eluted with 5 ml of 0.5 mol L^−1^ HNO_3_. Zinc isotopic compositions were analyzed by MC-ICPMS using standard bracketing techniques at the Institut de Physique du Globe de Paris (IPGP) following the procedures described in ref. (*100*). Zinc isotopic data are reported throughout the text using the δ^66^Zn_JMC-Lyon_ (‰) notation, the per mil deviation of the measured ^66^Zn/^64^Zn isotopic ratios relative to the JMC-Lyon standard

$$\delta^{66}\text{Zn}=\left[\frac{{\left(\frac{{\;}^{66}\text{Zn}}{{\;}^{64}\text{Zn}}\right)}_{\text{sample}}}{{\left(\frac{{\;}^{66}\text{Zn}}{{\;}^{64}\text{Zn}}\right)}_{\text{JMC}-\text{Lyon}}}-1\right]*1000$$

The USGS BHVO-2 reference material was purified for Zn following the same procedures as the sediments and rock samples and was subsequently analyzed to monitor the reproducibility and accuracy of the Zn isotopic data. Throughout this study, the average measured value of BHVO-2 was δ^66^Zn_JMC-Lyon_ = 0.30 ± 0.06‰ (2σ, *n* = 11), aligning with previously reported values (fig. S13). Sediment and rock samples analyzed in this study follow a mass fractionation line in terms of δ^66^Zn versus δ^68^Zn with a slope of ~2.3, suggesting negligible isobaric interferences in the measured isotopic ratios (fig. S13).

## Supplementary Materials

### The PDF file includes:

Supplementary Text S1 and S2 Figs. S1 to S13 Legends for data S1 and S2 References
