# Supplementary material for: Generation of carbonated peridotite melts via biogenic sediment recycling in modern subduction zones
Source: Sci Adv. 2026 May 6;12(19):eaec1599. doi: 10.1126/sciadv.aec1599 (PMC13148320; doi:10.1126/sciadv.aec1599)
Supplement: Supplementary file 1 — Supplementary Text S1 and S2 Figs. S1 to S13 Legends for data S1 and S2 References [file sciadv.aec1599_sm.pdf]

Science Advances
AAAS

Supplementary Materials for

Generation of carbonated peridotite melts via biogenic sediment recycling in modern subduction zones

Carlos Errázuriz-Henao *et al.*

Corresponding author: Carlos Errázuriz-Henao, cerrazuriz@unal.edu.co

Sci. Adv. **12**, eaec1599 (2026)
DOI: 10.1126/sciadv.aec1599

The PDF file includes:

Supplementary Text S1 and S2
Figs. S1 to S13
Legends for data S1 and S2
References

Other Supplementary Material for this manuscript includes the following:

Data S1 and S2

Supplementary Text

S1. Partial Melting models

To model the trace element concentration of Panama Basin (PB) metasedimentary restites, following partial melting at sub-arc depths, we used an inverted modal batch melting equation:

$$C_s = C_O \frac{D}{D+F(1-D)} \tag{1}$$

Where C_s is the concentration of an element in the residual solid, C_o the initial concentration of an element in the bulk sediment, *F* the degree of melting and *D* the bulk partition coefficient for each element.

Based on the near-trench bathymetry, as well as the influence of the subduction of the Malpelo rift and Carnegie ridge underneath the Magdalena Basanite-Nephelinite (MBN) suite, the average composition from the lower carbonated units was used as a potential subducted PB sediment. The residual mineralogy was chosen based on experimental datasets on carbonated sediment melting at sub-arc depths (3-4 GPa) and mantle wedge temperatures (>1200° C) (*64*, *65*). The modal percentage of each phase was set at 78 vol.% clinopyroxene, 15 vol.% carbonate, 5 vol.% garnet and 3 vol.% apatite, which are in line with previous estimates from sediment melting modelling for the colombian arc-front (*33*). Mineral-melt partition coefficients (K_d) in equilibrium with evolve compositions were obtained from the Earth Reference Data and Models (*Earthref*) database for clinopyroxene and garnet, and from ref. (*65*, *106*) for carbonate and apatite, respectively. Based on recent estimates from other cordilleran arcs (*107*), a PB metasedimentary restitic composition following 45% of partial melting was set to represent the potential carbonated contributor for the colombian rear-arc region (see supplementary Fig. S7).

The modified mantle source of MBN was then computed by considering a solid-solid bulk mixing of 5%, 10% and 15% of metasedimentary restite with an ambient peridotitic mantle. The partial melts from this modified mantle where model using a modal batch melting equation:

$$C_l = \frac{C_o}{D+F(1-D)} \tag{2}$$

Where C_l is the composition of an element in the melt, C_o the initial concentration of an element in the modified mantle source, *F* the degree of melting and *D* the bulk partition coefficient for each element.

To align with the relative enrichment of a rear-arc mantle compared to the typically depleted mantle wedge expected beneath arc-fronts (*108*, *109*), a trace element enriched mantle was used as the unmodified peridotitic source (see supplementary Fig. S12). The mantle composition is taken from ref. (*107*). Partial melts from a modified mantle wedge were modeled using a phlogopite-lherzolite composition comprising 46 vol.% olivine, 30 vol.% clinopyroxene, 15 vol.% garnet, 8 vol.% orthopyroxene, and 1 vol.% phlogopite.

K_d for olivine, clinopyroxene, garnet, and orthopyroxene in equilibrium with carbonated silicate melts were obtained from ref. (*48*). For rare earth elements (REE) elements not specifically listed, an average of neighboring elements partition coefficients was used. Partition coefficients for phlogopite were taken from ref. (*110*). The modeled partial melts shown in Figure 6, and supplementary Figure S8 and S10 for unmodified carbonated-silicate melts were computed using the same unmodified mantle composition, mineral proportions, and partition coefficients described above. Partial melts from non-carbonated sources were calculated using the same mantle composition as the other models but employed mineral-melt partition coefficients derived from CO_2- and carbonate-free experimental datasets in equilibrium with basaltic melt from ref. (*111*), and complemented by data from the *Earthref* database. Phlogopite was excluded from the mineral modes in calculations for partial melts from non-carbonated mantle sources.

S2. Zn isotopic melting models

The Zn isotopic effects following partial melting of a carbonate modified mantle lithology were modelled following previous published procedures and modify to accommodate for modal batch melting (*10*, *112*).

$$C_l^i = \frac{C_o^i}{\left(D*\alpha Zn^{\frac{i}{j}}_{b-m}\right)+F\left(D*\alpha Zn^{\frac{i}{j}}_{b-m}\right)} \tag{3}$$

Where C_l^i and C_o^i is the concentration of isotope i (i.e ^{66}Zn) in the melt and source, respectively, and $\alpha Zn^{\frac{i}{j}}_{1-2}$ is fractionation factor (α) at 1573 K of isotope ${}^{i}Zn$ to isotope ${}^{j}Zn$ in phase b (bulk) relative to phase m (melt). Unmodified mantle Zn isotopic composition was assumed to be that of the bulk mantle peridotite (*42*). Mineral-melt isotopic fractionation factors were taken from ref. (*113*). We adopt the mineral-melt isotope fractionation factor of phlogopite to be the same as that of olivine based on ionic inter-mineral fractionation models (*114*).

We set the Zn isotopic composition of the PB metasedimentary restites to be the same as the most $CaCO_3$-rich sediment sample. This is assumed based on the high compatibility of Zn (>1) during sediment partial melting, which implies little isotopic fractionation in the residue. Our Zn isotopic model does not account for potential oceanic crust dehydration reactions, which could mobilize isotopically heavy Zn–SO_4^{2-} complexes during the early stages of subduction (*115*). However, this process is likely to have only a minor impact, especially in warm subduction zones, where mantle metasomatism in the arc-front is primarily driven by sediment melts rather than sediment-derived or altered oceanic igneous crust fluids at sub-arc depths (*116*). Additionally, due to the limited stability field of serpentinite at high P-T conditions (>160 km, 700 °C) (*117*), the involvement of isotopically heavy Zn fluids derived from serpentinite is unlikely under the melt generation conditions (~200 km) beneath the Magdalena Volcanic Field (MVF).

Fig. S1.

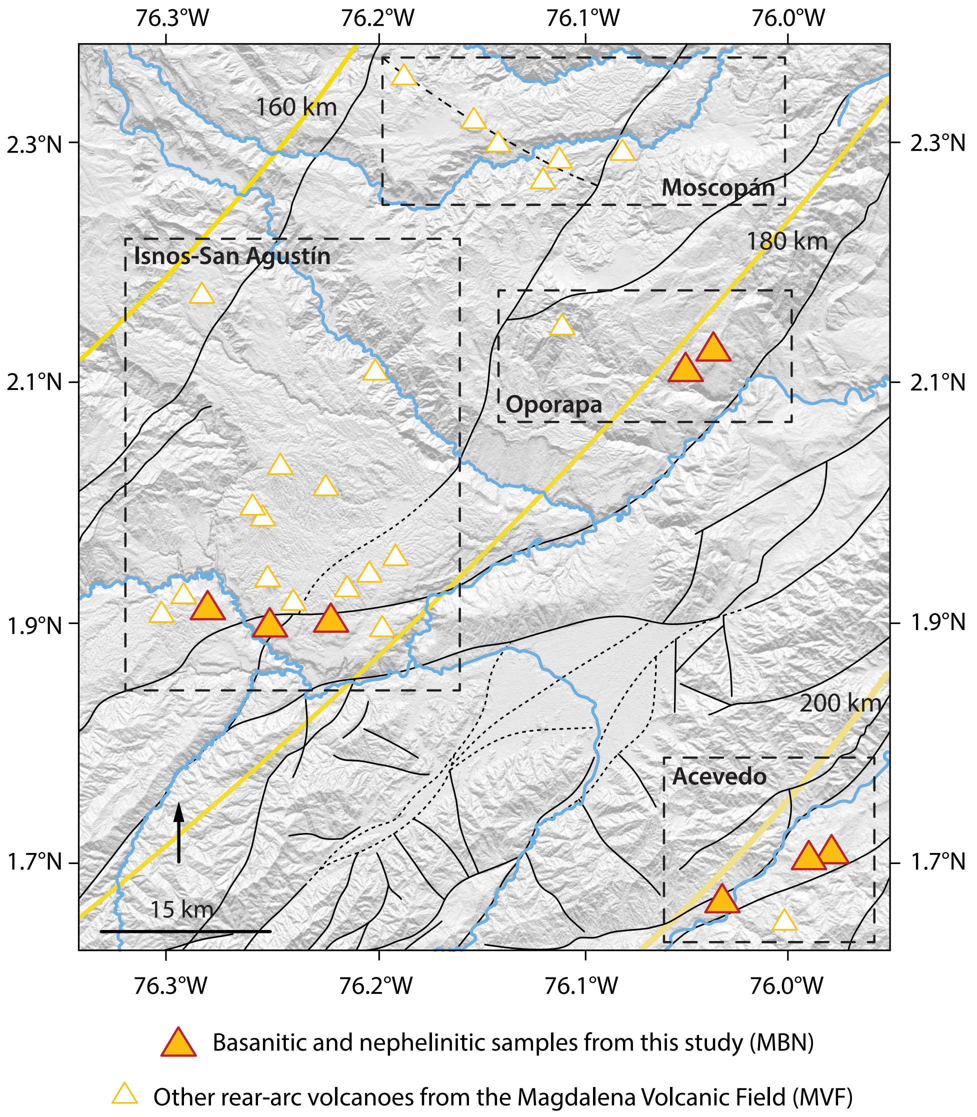

Local map of the study area. Distribution of the different volcanic clusters within the Magdalena Volcanic Field (MVF) and the location of the samples analyzed in this study. Detailed descriptions of the individual volcanic structures can be found in ref. (*22*). For exact coordinates please refer to Supplementary Data S2.

Fig. S2.

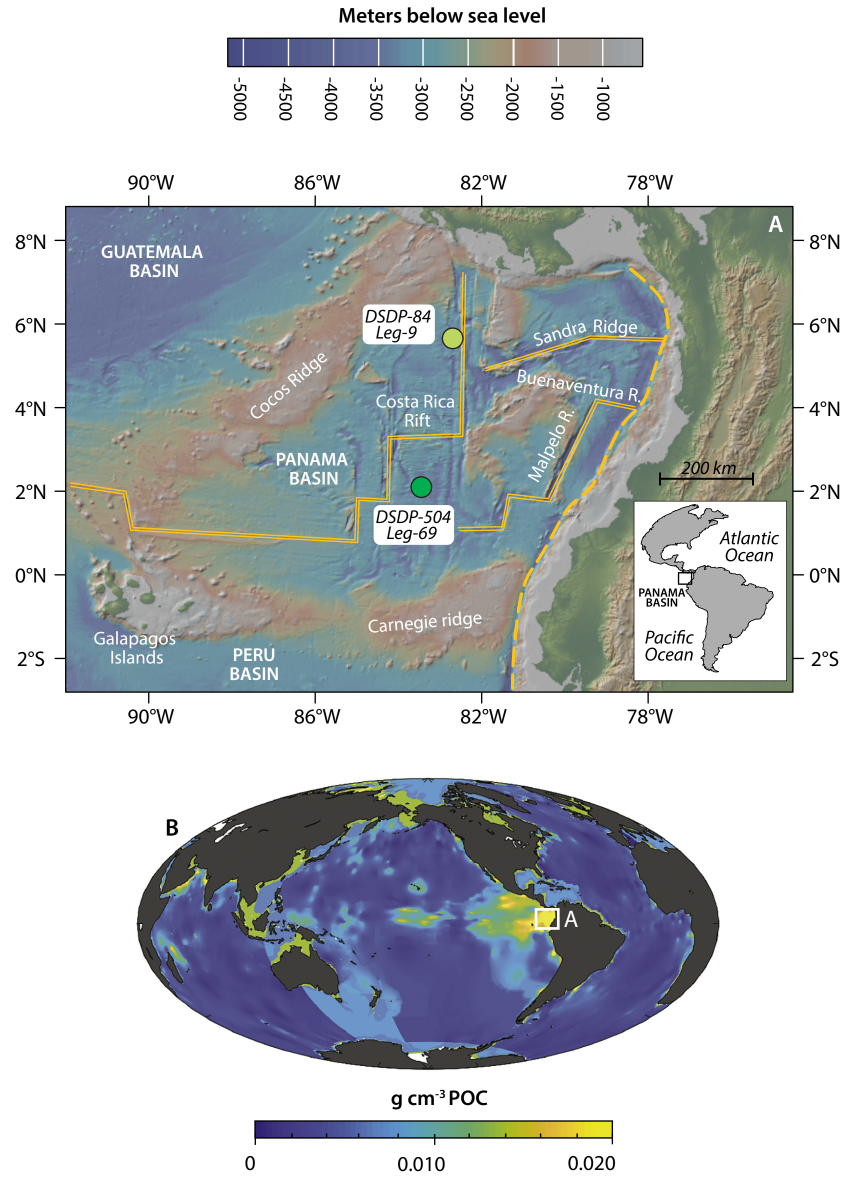

Bathymetry, tectonic setting, and oceanographic conditions of the Panama Basin (PB). (A) Bathymetric map and major tectonic features of the PB, including the Cocos and Carnegie Ridges, which separate the PB from the rest of the Pacific Ocean. The locations of Deep-Sea Drilling Project (DSDP) sites 84 and 504 are shown for reference. Sediment thickness near the trench reaches ~800 m according to seismic studies (*83*). **(B)** Particulate organic carbon concentrations at the seafloor surface highlight the global maximum in the PB, reflecting both high carbon export and high primary productivity (*118*). Figure reprinted from Earth. Sci. Rev. 204, 103146. D. E. LaRowe, S. Arndt, J. A. Bradley, E. R. Estes, A. Hoarfrost, S. Q. Lang, K. G. Lloyd, N. Mahmoudi, W. D. Orsi, S. R. Shah Walter, A. D. Steen, R. Zhao, The fate of organic carbon in marine sediments - New insights from recent data and analysis. Copyright (2020) with permission from Elsevier.

Fig. S3.

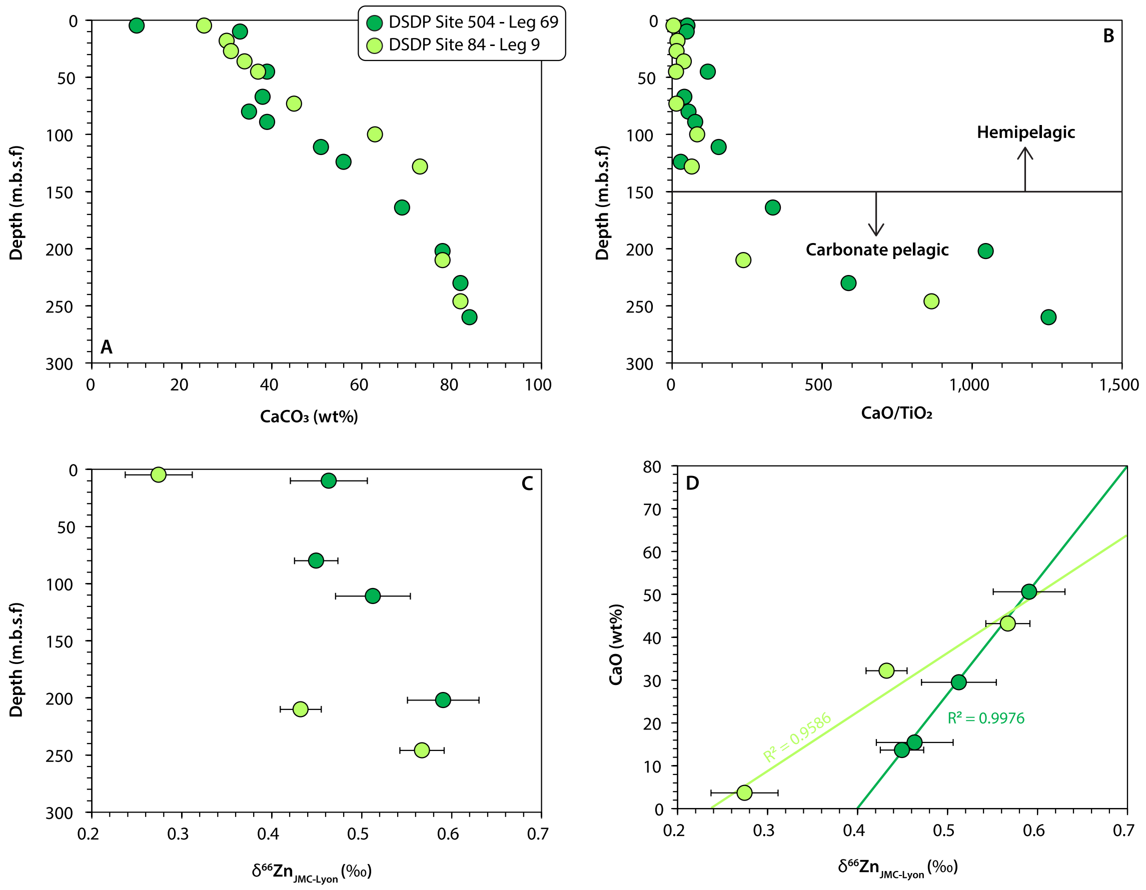

Composition of Panama Basin DSDP sediment samples from this study. (A-B-C) Core depth in meters below the sea floor (m.b.s.f.) plotted against carbonate content ($CaCO_3$), Ca/TiO_2 ratios and measured $\delta^{66}Zn$ isotopic ratios (see supplementary Data S1). $CaCO_3$ contents increase consistently downcore, but the terrigenous fraction decreases rapidly at ~150 m.b.s.f, marking the limit between the hemipelagic and carbonate pelagic sequence. $\delta^{66}Zn$ isotopic ratios positively correlate with depth for individual DSDP sites. **(D)** At a given depth and CaO concentration, sediments from DSDP site 504 show a relatively heavier Zn isotopic composition compared to DSDP site 84. However, both sites showcase a remarkably good correlation between CaO concentrations and $\delta^{66}Zn$. CaO concentrations are depicted here because they were directly analyzed from the same sample powder, whereas $CaCO_3$ concentrations are derived from shipboard analyses (*119*, *120*). For a complete trace, major and radiogenic isotopic composition of both DSDP sites sediment samples refer to ref. (*21*).

Fig. S4.

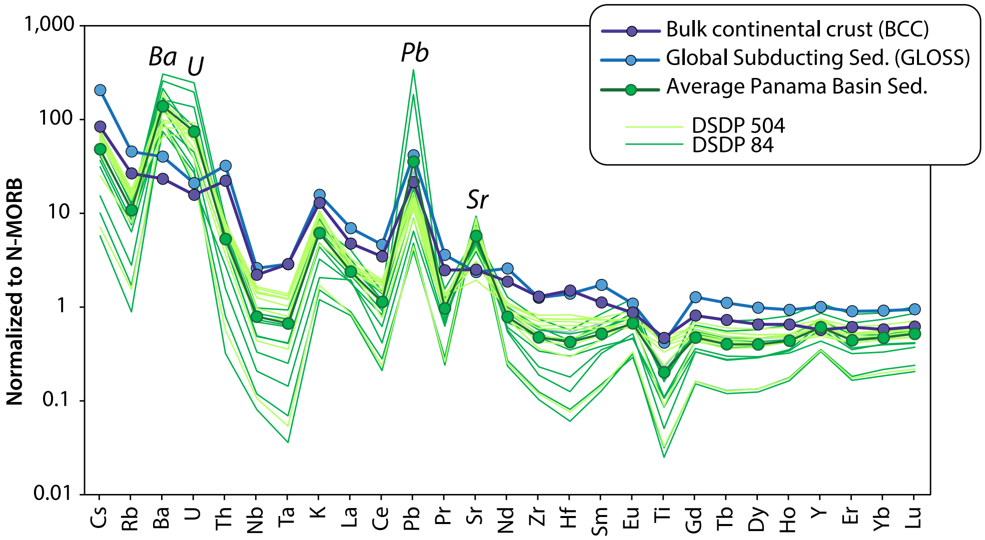

Normalized trace element composition of Panama Basin sediments. Complete array of trace elements from DSDP sites 504 and 84, as well as the average composition of PB sediment from both drill sites. Composition of the Bulk Continental Crust (BCC) (*32*) and Global Subducting Sediments (GLOSS) (*13*) are also shown for comparison. The enrichments in Ba and U in Panama Basin sediments signal the intense contribution of biogenic components (barite and uraninite), whereas the enrichment in Sr is related to the proportion of carbonates (calcite). Their lower concentrations of high-field strength elements (HFSE) and REE when compared with the BCC and GLOSS is directly related to the terrigenous dilution, especially in the most carbonate-rich samples.

Fig. S5.

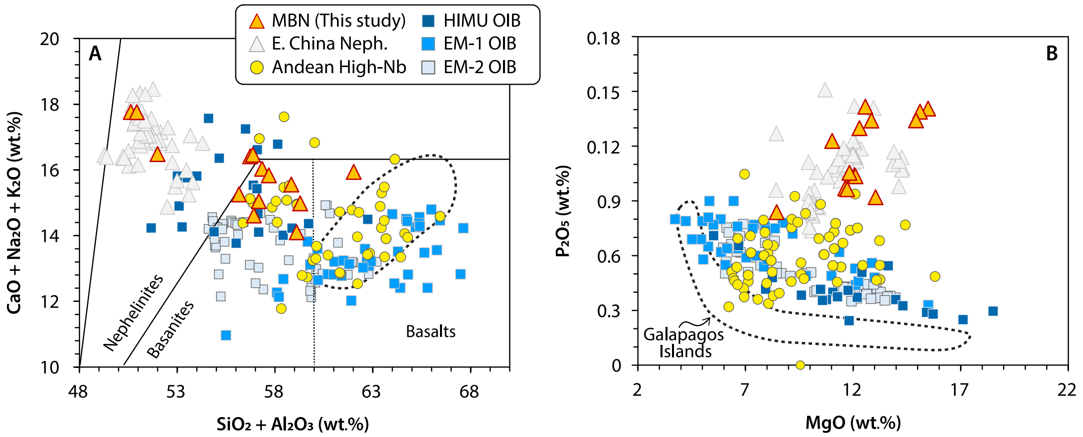

Additional major element systematics of MBN rocks and other mantle-derived magmas. (A) Classification scheme for alkaline rocks (*121*) showcasing the nephelinitic nature of some MBN lavas and their close association with HIMU and Eastern China nephelinites. **(B)** MgO (wt.%) plotted against P_2O_5 (wt.%) displaying the contrasting trends between MBN and Eastern China nephelinites, OIB-type rocks (i.e. HIMU, EM-1, EM-2 and Galapagos) and Andean high-Nb basalts. Regardless of the MgO concentrations, MBN shows higher P_2O_5 contents than all other series considered. For data sources please refer to Figure 3 in the main text.

Fig. S6.

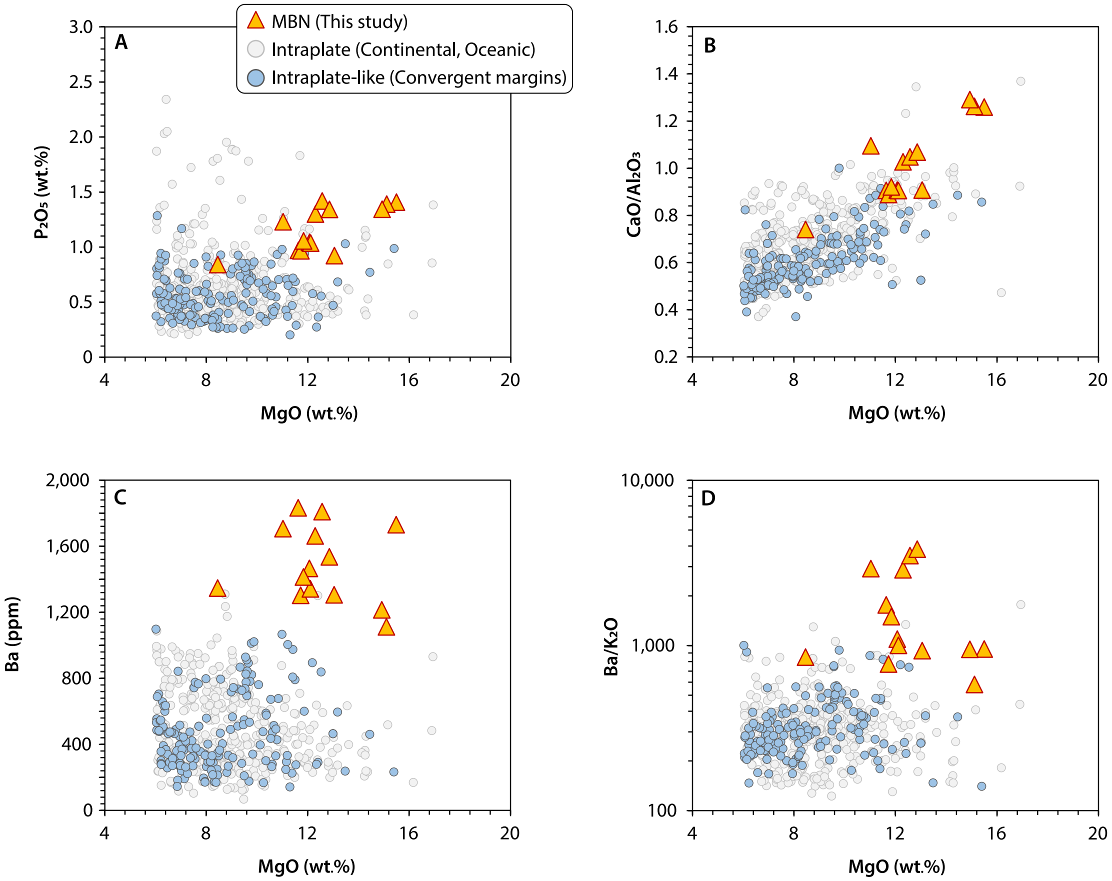

Global comparison between MBN lavas and intraplate-like rocks from convergent margins, continental interiors, and oceanic basins. The comparison shown here is based on the global compilation of high-Nb basalts from ref. (*40*), which relies exclusively on high-resolution GEOROC whole-rock data. The dataset was filtered to include mantle-derived basalts, basanites, and nephelinites with primitive-mantle values of Ce/Pb, Th/Nb, and U/Nb, along with high-MgO compositions (>6 wt.%). These criteria minimize the inclusion of samples with clear arc-like signatures or evolved, fractionated melts. Only Quaternary samples are shown. **(A-B)** Although some intraplate rocks from continental interiors and oceanic basins show elevated P_2O_5 and high CaO/Al_2O_3 ratios, intraplate-like rocks from other convergent margins do not consistently overlap with the MBN. **(C-D)** Ba enrichment in MBN lavas stands out globally, particularly given their primitive high-MgO compositions. Ba/K_2O ratios in MBN lavas also appear to reach a global maximum.

Fig. S7.

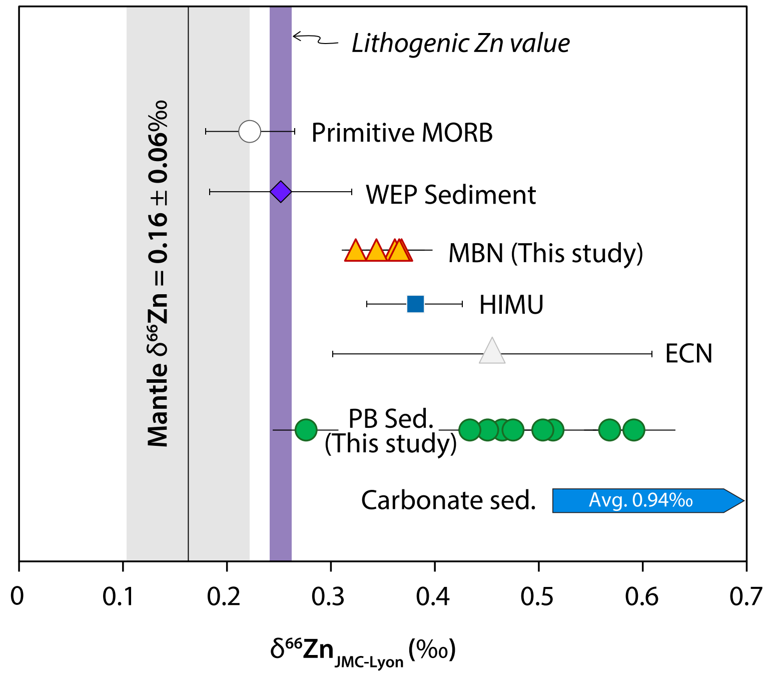

Stable Zn isotopic composition of MBN rocks and PB sediments. The gray field represents the mantle peridotite Zn isotopic composition (*42*). The lithogenic Zn value is taken from ref. (*30*), denoting the average of several surface terrigenous reservoirs. MORB compositions from ref. (*41*), Eastern China nephelinites (ECN) from ref. (*12*) and HIMU from ref. (*10*). Western Equatorial Pacific (WEP) sediments are taken from ref. (*29*) and other deep-sea carbonates, including samples from the Eastern Equatorial Pacific (EEP) are taken from ref. (*28*).

Fig. S8.

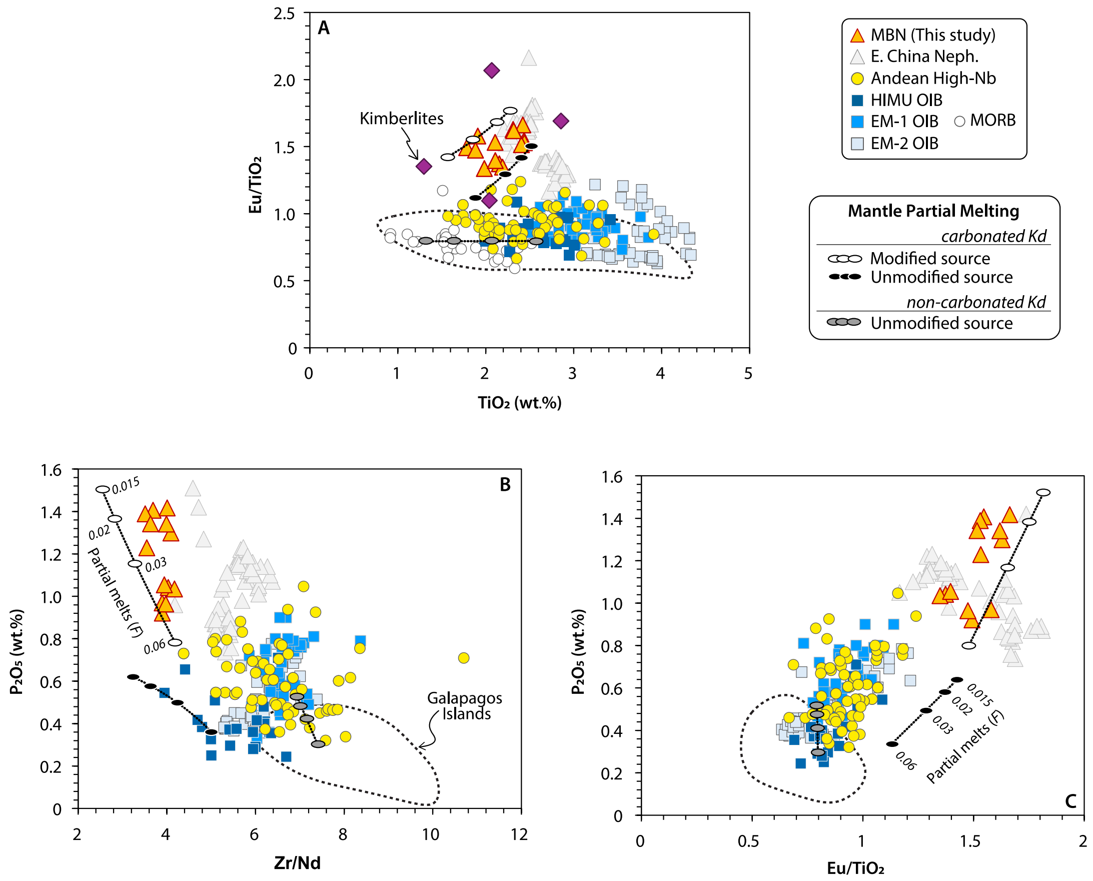

Carbonate-sensitive geochemical proxies in MBN lavas. (A) TiO_2 (wt.%) vs Eu/TiO_2 ratio showing the strong decoupling imposed by MBN rocks, Eastern China nephelinites and kimberlitic melts from the mantle array represented by MORB and other OIB-type lavas (for data sources please refer to Figure 3 in main text). **(B-C)** Covariations between carbonated-sensitive geochemical proxies (Eu/TiO_2 and Zr/Nd, see main text) and P_2O_5 (wt.%) contents. The good correlations observed in MBN rocks agree with a variably modified mantle source by carbonated and apatite-bearing components. Partial melting models (see Supplementary Information S2) describe the evolution of modified (with variable proportion of PB metasedimentary restites) and unmodified mantle melts using partition coefficients (K_d) in equilibrium with a carbonated peridotite (*48*). Unmodified mantle partial melts using K_d in equilibrium with non-carbonated lithologies are also shown for comparison. Note that unmodified carbonated peridotite mantle melts reproduce the high Eu/TiO_2 and low Zr/Nd ratios but fail to account for the high P_2O_5 content of MBN and Eastern China nephelinites. This argues for both a carbonate-rich and apatite-bearing contributors in their mantle sources. For further details see Supplementary Text S1.

Fig. S9.

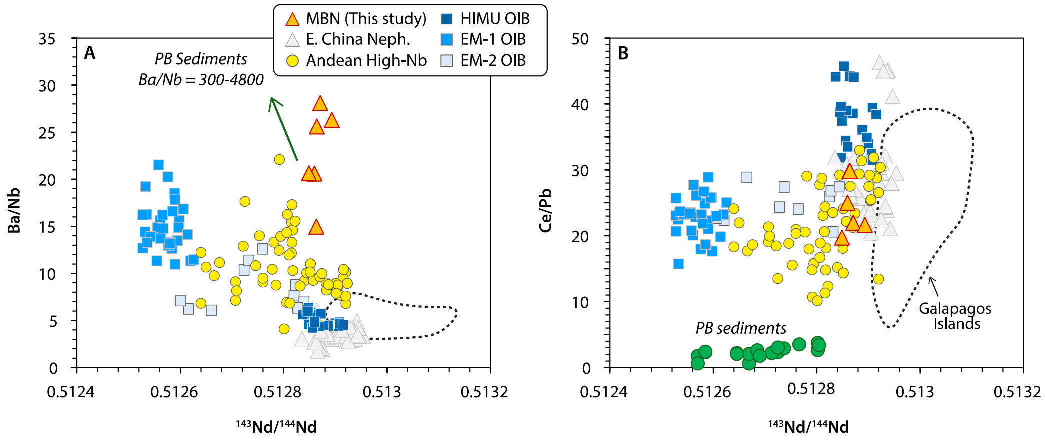

Relationships between $^{143}Nd/^{144}Nd$, Ba/Nb and Ce/Pb ratios. (A-B) MBN rocks show no correlation between crustal-sensitive ratios such as Ba/Nb and Ce/Pb with $^{143}Nd/^{144}Nd$ isotope ratios. This suggests a negligible contribution from continental materials in their mantle source, either as crustal contaminants or from EM-1 and EM-2-type mantle sources (*50*).

Fig. S10.

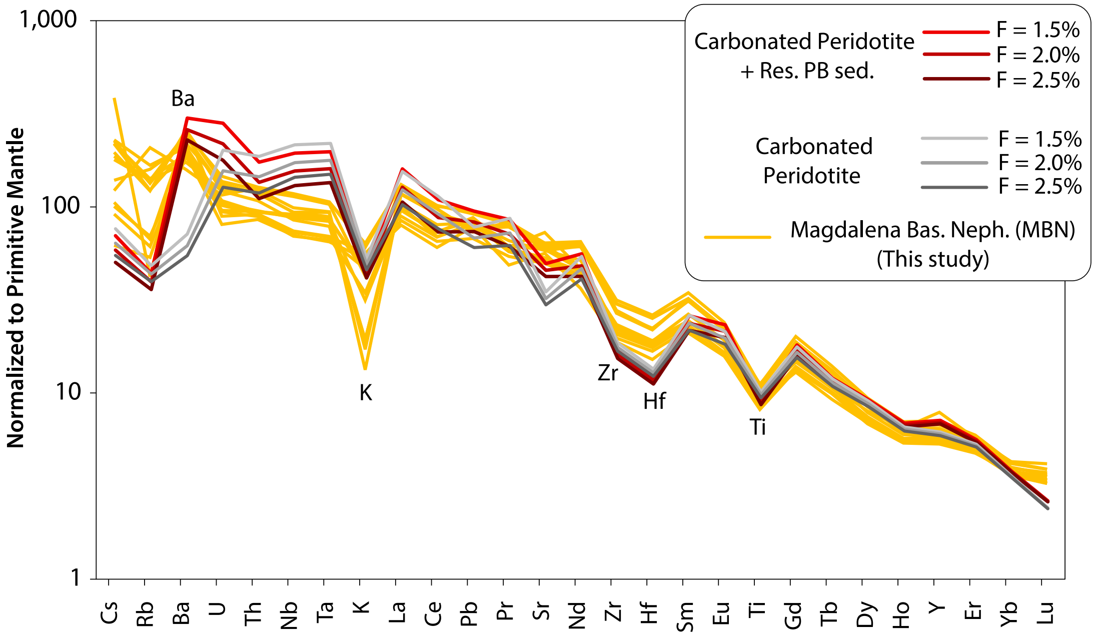

Model results for a complete array of trace elements. Modeled partial melts were derived from a modified mantle source with a phlogopite-lherzolite composition, consisting of 46 vol.% olivine, 30 vol.% clinopyroxene, 15 vol.% garnet, 8 vol.% orthopyroxene, and 1 vol.% phlogopite. The proportion of PB metasedimentary restites mixed with an unmodified mantle composition was set at 10%, representing the intermediate values of the models shown in Fig. 6 and Fig. 7 in the main text. Modeled partial melts from an unmodified mantle source were computed using the same partition coefficients, mineral proportions, and mantle composition as in the modified melting model.

Fig. S11.

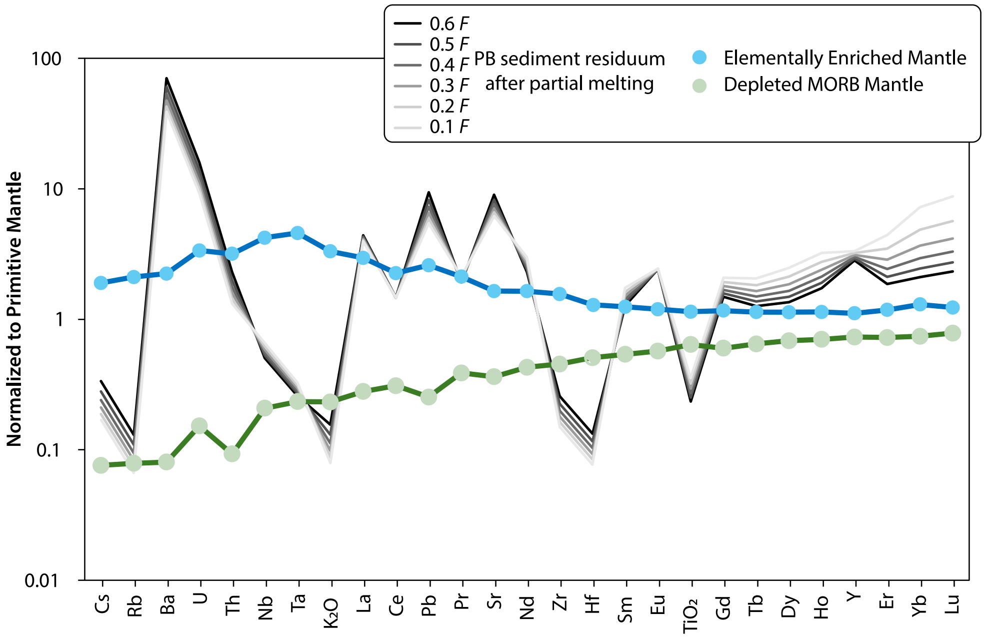

Composition of the mantle component and PB metasedimentary restites following partial melting. PB metasedimentary restitic composition calculated from an inverse batch melting model. The modal percentage of each phase was set at 78 vol.% clinopyroxene, 15 vol.% carbonate, 5 vol.% garnet and 3 vol.% apatite. A restitic composition after 45% partial melting was used as the carbonated contributor in the model calculation depicted in Figure 6 and 7, and Figure S8 and S10. A trace element enriched mantle was used as the unmodified peridotite source. This mantle composition is based on an intraplate-like lava from the Trans-Mexican Volcanic Belt with a remarkably flat normalized trace element pattern and negligible signs of subduction influence, considered as a potential background mantle underneath thick cordilleran arc sections (*107*). A Depleted MORB mantle (DMM) (*122*) is shown for comparison.

Fig. S12.

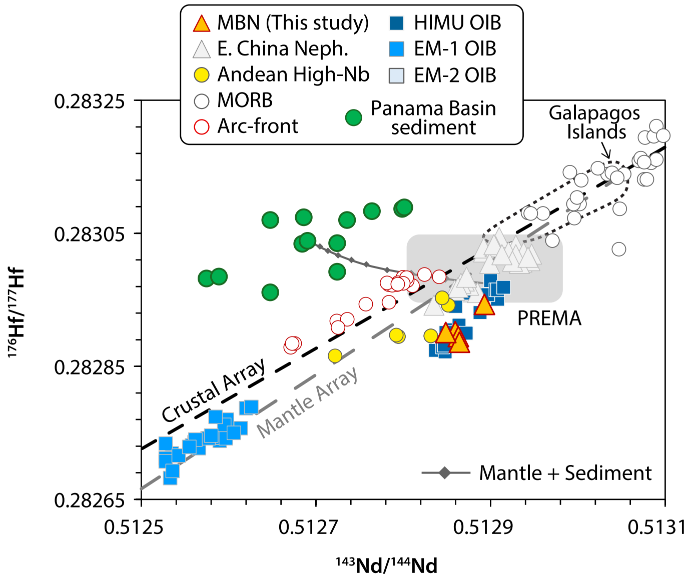

$^{143}Nd/^{144}Nd$ vs. $^{176}Hf/^{177}Hf$ isotopic systematics of MBN rocks. MBN rocks display a similar composition to those found in HIMU-type OIB from St. Helena and Cook-Austral Islands and partially overlap with at least one sample from Andean high-Nb basalts. The latter is part of the Argentinean back-arc basalts for which a slight Nd-Hf decoupling lower than the mantle array has been observed (*123*). In contrast to Pb and Sr isotopes, for which the metasedimentary restites strongly control the elemental Pb and Sr budget in modified mantle lithologies, Hf and Nd isotopes are mostly dominated by the unmodified mantle source. In this sense, the Nd-Hf isotopic composition of MBN lavas likely represent the most accurate representation of the unmodified mantle beneath the Colombian rear-arc region.

Fig. S13.

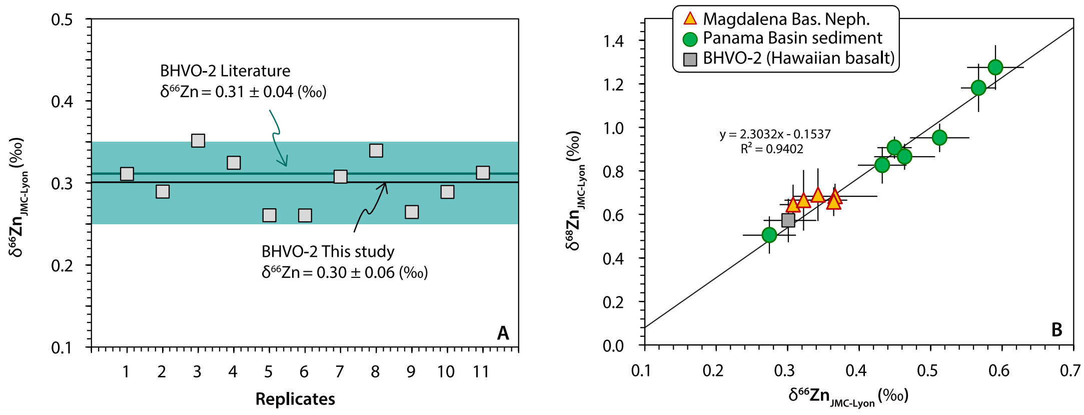

Stable Zn isotopic composition of measured standards and covariation between δ^{66}Zn and δ^{68}Zn in analyzed samples in this study. (A) An aliquot of BHVO-2 international standard, purified following the same procedure as the rock samples (see Methods for details) was measured 11 times during two analytical sessions, resulting in an average of δ^{66}Zn = 0.30 ± 0.06 ‰ (2SD, n = 11). This aligns with the accepted value and external reproducibility from published literature (e.g., ref. (*124*), δ^{66}Zn = 0.28 ± 0.04 ‰), as compiled from the Geological and Reference Materials Data Base (GEOREM). **(B)** δ^{66}Zn plotted against δ^{68}Zn in analyzed samples showcasing the mass dependent fractionation line.

Data S1. (separate file)
Zinc isotopic composition of Panama Basin sediments from the Eastern Equatorial Pacific.

Data S2. (separate file)
Bulk rock major, trace and Sr, Pb, Nd, Hf and Zn isotopic composition of MBN lavas.

REFERENCES

1. R. D. Müller, B. Mather, A. Dutkiewicz, T. Keller, A. Merdith, C. M. Gonzalez, W. Gorczyk, S. Zahirovic, Evolution of Earth's tectonic carbon conveyor belt. *Nature* **605**, 629–639 (2022).

2. A. Dutkiewicz, R. D. Müller, J. Cannon, S. Vaughan, S. Zahirovic, Sequestration and subduction of deep-sea carbonate in the global ocean since the Early Cretaceous. *Geology* **47**, 91–94 (2019).

3. R. Dasgupta, M. M. Hirschmann, The deep carbon cycle and melting in Earth's interior. *Earth Planet. Sci. Lett.* **298**, 1–13 (2010).

4. P. B. Kelemen, C. E. Manning, Reevaluating carbon fluxes in subduction zones, what goes down, mostly comes up. *Proc. Natl. Acad. Sci. U.S.A.* **112**, E3997–E4006 (2015).

5. T. Plank, C. E. Manning, Subducting carbon. *Nature* **574**, 343–352 (2019).

6. F. Arzilli, M. Burton, G. La Spina, C. G. Macpherson, P. E. van Keken, J. McCann, Decarbonation of subducting carbonate-bearing sediments and basalts of altered oceanic crust: Insights into recycling of CO_2 through volcanic arcs. *Earth Planet. Sci. Lett.* **602**, 117945 (2023).

7. G. Zeng, L. H. Chen, A. W. Hofmann, X. J. Wang, J. Q. Liu, X. Yu, L. W. Xie, Nephelinites in eastern China originating from the mantle transition zone. *Chem. Geol.* **576**, 120276 (2021).

8. A. Giuliani, R. N. Drysdale, J. D. Woodhead, N. J. Planavsky, D. Phillips, J. Hergt, W. L. Griffin, S. Oesch, H. Dalton, G. R. Davies, Perturbation of the deep-Earth carbon cycle in response to the Cambrian Explosion. *Sci. Adv.* **8**, eabj1325 (2022).

9. S. E. Mazza, E. Gazel, M. Bizimis, R. Moucha, P. Béguelin, E. A. Johnson, R. J. McAleer, A. V. Sobolev, Sampling the volatile-rich transition zone beneath Bermuda. *Nature* **569**, 398–403 (2019).

10. X. Y. Zhang, L. H. Chen, X. J. Wang, T. Hanyu, A. W. Hofmann, T. Komiya, K. Nakamura, Y. Kato, G. Zeng, W. X. Gou, W. Q. Li, Zinc isotopic evidence for recycled carbonate in the deep mantle. *Nat. Commun.* **13**, 6085 (2022).

11. S. A. Liu, Y. R. Qu, Z. Z. Wang, M. L. Li, C. Yang, S. G. Li, The fate of subducting carbon tracked by Mg and Zn isotopes: A review and new perspectives. *Earth. Sci. Rev.* **228**, 104010 (2022).

12. S. A. Liu, Z. Z. Wang, S. G. Li, J. Huang, W. Yang, Zinc isotope evidence for a large-scale carbonated mantle beneath eastern China. *Earth Planet. Sci. Lett.* **444**, 169–178 (2016).

13. T. Plank, "The chemical composition of subducting sediments" in *Treatise on Geochemistry*, H. D. Holland, K. K. Turekian, Eds., (Elsevier, ed. 2, 2014), vol. 4, pp. 607–629.

14. R. Dasgupta, A. Mallik, K. Tsuno, A. C. Withers, G. Hirth, M. M. Hirschmann, Carbon-dioxide-rich silicate melt in the Earth's upper mantle. *Nature* **493**, 211–215 (2013).

15. P. D. Clift, A revised budget for Cenozoic sedimentary carbon subduction. *Rev. Geophys.* **55**, 97–125 (2017).

16. C. Errázuriz-Henao, A. Gómez-Tuena, M. Parolari, M. Weber, Climate-driven compositional modifications of arc volcanoes along the East Equatorial Pacific Margin—The magmatic response to a cooling planet. *Earth. Sci. Rev.* **234**, 104228 (2022).

17. J. T. Pennington, K. L. Mahoney, V. S. Kuwahara, D. D. Kolber, R. Calienes, F. P. Chavez, Primary production in the eastern tropical Pacific: A review. *Prog. Oceanogr.* **69**, 285–317 (2006).

18. K. Tsuno, R. Dasgupta, L. Danielson, K. Righter, Flux of carbonate melt from deeply subducted pelitic sediments: Geophysical and geochemical implications for the source of Central American volcanic arc. *Geophys. Res. Lett.* **39**, L16307 (2012).

19. M. A. Gutscher, J. Malavieille, S. Lallemand, J. Y. Collot, Tectonic segmentation of the North Andean margin: Impact of the Carnegie Ridge collision. *Earth Planet. Sci. Lett.* **168**, 255–270 (1999).

20. R. McGirr, M. Seton, S. Williams, Kinematic and geodynamic evolution of the Isthmus of Panama region: Implications for Central American Seaway closure. *Bull. Geol. Soc. Am.* **133**, 867–884 (2021).

21. C. Errázuriz-Henao, A. Gómez-Tuena, M. Parolari, M. Weber, A biogeochemical imprint of the Panama Basin in the North Andean Arc. *Geochem. Geophys. Geosyst.* **22**, e2021GC009835 (2021).

22. M. L. Monsalve-Bustamante, J. Gómez, A. Nuñez-Tello, "Rear arc small–volume basaltic volcanism in Colombia: Monogenetic volcanic fields" in *The Geology of Colombia, Volume 4 Quaternary*, J. Gómez, A. O. Pinilla-Pachon, Eds. (Servicio Geologico Colombiano, 2020), pp. 353–396.

23. J. Idárraga-García, J. M. Kendall, C. A. Vargas, Shear wave anisotropy in northwestern South America and its link to the Caribbean and Nazca subduction geodynamics. *Geochem. Geophys. Geosyst.* **17**, 3655–3673 (2016).

24. E. E. Rodríguez, S. L. Beck, M. Ruiz, A. Meltzer, D. E. Portner, S. Hernández, M. Segovia, H. Agurto-Detzel, P. Charvis, Seismic imaging of the Northern Andean subduction zone from teleseismic tomography: A torn and fragmented Nazca slab. *Geophys. J. Int.* **236**, 593–606 (2024).

25. S. M. Straub, A. Gómez-Tuena, P. Vannucchi, Subduction erosion and arc volcanism. *Nat. Rev. Earth Environ.* **1**, 574–589 (2020).

26. S. C. Carter, A. Paytan, E. M. Griffith, Toward an improved understanding of the marine barium cycle and the application of marine barite as a paleoproductivity proxy. *Minerals* **10**, 421 (2020).

27. V. Mavromatis, A. G. González, M. Dietzel, J. Schott, Zinc isotope fractionation during the inorganic precipitation of calcite—Towards a new pH proxy. *Geochim. Cosmochim. Acta* **244**, 99–112 (2019).

28. S. Pichat, C. Douchet, F. Albarède, Zinc isotope variations in deep-sea carbonates from the eastern equatorial Pacific over the last 175 ka. *Earth Planet. Sci. Lett.* **210**, 167–178 (2003).

29. Y. R. Qu, S. A. Liu, H. Wu, M. L. Li, H. C. Tian, Tracing carbonate dissolution in subducting sediments by zinc and magnesium isotopes. *Geochim. Cosmochim. Acta* **319**, 56–72 (2022).

30. S. H. Little, D. Vance, J. McManus, S. Severmann, Key role of continental margin sediments in the oceanic mass balance of Zn and Zn isotopes. *Geology* **44**, 207–210 (2016).

31. M. Kunzmann, G. P. Halverson, P. A. Sossi, T. D. Raub, J. L. Payne, J. Kirby, Zn isotope evidence for immediate resumption of primary productivity after snowball Earth. *Geology* **41**, 27–30 (2013).

32. R. L. Rudnick, S. Gao, "Composition of the continental crust" in *Treatise on Geochemistry*, H. D. Holland, K. K. Turekian, Eds., (Elsevier, ed. 2, 2014), vol. 4, pp. 1–51.

33. C. Errázuriz-Henao, A. Gómez-Tuena, J. Duque-Trujillo, M. Weber, The role of subducted sediments in the formation of intermediate mantle-derived magmas from the Northern Colombian Andes. *Lithos* **336–337**, 151–168 (2019).

34. T. Plank, V. Balzer, M. Carr, Nicaraguan volcanoes record paleoceanographic changes accompanying closure of the Panama gateway. *Geology* **30**, 1087–1090 (2002).

35. J. Lages, Z. Chacón, V. Burbano, L. Meza, S. Arellano, M. Liuzzo, G. Giudice, A. Aiuppa, M. Bitetto, C. López, Volcanic gas emissions along the colombian arc segment of the northern volcanic zone (CAS-NVZ): Implications for volcano monitoring and volatile budget of the Andean Volcanic Belt. *Geochem. Geophys. Geosyst.* **20**, 5057–5081 (2019).

36. M. Marín-Cerón, H. Leal-Mejía, M. Bernet, J. Mesa-García, "Late Cenozoic to modern-day volcanism in the Northern Andes: A geochronological, petrographical, and geochemical review" in *Geology and Tectonics of Northwestern South America*, F. Cediel, R. P. Shaw, Eds. (Springer, 2019), pp. 603–648.

37. J. S. Jaramillo-Ríos, A. Cardona, S. Zapata, V. Valencia, G. Monsalve, J. Vervoort, A mantle origin for Pliocene SiO_2-rich ignimbrites in the modern Colombian magmatic arc. *Lithos* **480–481**, 107666 (2024).

38. S. Kroonenberg, H. Pichler, C. Schmitt-Riegraf, Young alkali-basaltic to nephelinitic volcanism in the southern Colombian Andes-Origin by subduction of a spreading rift? *Zbl. Geol. PaHiont.* **1**, 919–936 (1987).

39. A. Giuliani, D. Graham Pearson, A. Soltys, H. Dalton, D. Phillips, S. F. Foley, E. Lim, K. Goemann, W. L. Griffin, R. H. Mitchell, Kimberlite genesis from a common carbonate-rich primary melt modified by lithospheric mantle assimilation. *Sci. Adv.* **6**, 1–9 (2020).

40. R. Cai, J. Liu, D. G. Pearson, A. Giuliani, P. E. van Keken, S. Oesch, Widespread PREMA in the upper mantle indicated by low-degree basaltic melts. *Nat. Commun.* **14**, 8150 (2023).

41. P. Sun, Y. Niu, M. Duan, S. Chen, P. Guo, H. Gong, Y. Xiao, X. Wang, Zinc isotope fractionation during mid-ocean ridge basalt differentiation: Evidence from lavas on the East Pacific Rise at 10°30′N. *Geochim. Cosmochim. Acta* **346**, 180–191 (2023).

42. P. A. Sossi, O. Nebel, H. S. C. O'Neill, F. Moynier, Zinc isotope composition of the Earth and its behaviour during planetary accretion. *Chem. Geol.* **477**, 73–84 (2018).

43. Y. Weiss, C. Class, S. L. Goldstein, T. Hanyu, Key new pieces of the HIMU puzzle from olivines and diamond inclusions. *Nature* **537**, 666–670 (2016).

44. N. V. Vladykin, F. Pirajno, Types of carbonatites: Geochemistry, genesis and mantle sources. *Lithos* **386–387**, 105982 (2021).

45. M. L. Gorring, S. M. Kay, Carbonatite metasomatized peridotite xenoliths from southern Patagonia: Implications for lithospheric processes and Neogene plateau magmatism. *Contrib. Mineral. Petrol.* **140**, 55–72 (2000).

46. R. L. Rudnick, W. F. McDonough, B. W. Chappell, Carbonatite metasomatism in the northern Tanzanian mantle: Petrographic and geochemical characteristics. *Earth Planet. Sci. Lett.* **114**, 463–475 (1993).

47. D. A. Ionov, C. Dupuy, S. Y. O'Reilly, M. G. Kopylova, Y. S. Genshaft, Carbonated peridotite xenoliths from Spitsbergen: Implications for trace element signature of mantle carbonate metasomatism. *Earth Planet. Sci. Lett.* **119**, 283–297 (1993).

48. R. Dasgupta, M. M. Hirschmann, W. F. McDonough, M. Spiegelman, A. C. Withers, Trace element partitioning between garnet lherzolite and carbonatite at 6.6 and 8.6 GPa with applications to the geochemistry of the mantle and of mantle-derived melts. *Chem. Geol.* **262**, 57–77 (2009).

49. A. W. Hofmann, C. Class, "Canonical trace element ratios in oceanic basalts" in *Treatise on Geochemistry*, A. D. Anbar, D. Weis, Eds., (Elsevier, ed. 3, 2025), vol. 1, pp. 565–589.

50. M. Willbold, A. Stracke, Trace element composition of mantle end-members: Implications for recycling of oceanic and upper and lower continental crust. *Geochem. Geophys. Geosyst.* **7**, Q04004 (2006).

51. S. J. Turner, C. H. Langmuir, M. A. Dungan, S. Escrig, The importance of mantle wedge heterogeneity to subduction zone magmatism and the origin of EM1. *Earth Planet. Sci. Lett.* **472**, 216–228 (2017).

52. E. S. Steenstra, M. Klaver, J. Berndt, S. Flemetakis, A. Rohrbach, S. Klemme, Thermal stability of F-rich phlogopite and K-richterite during partial melting of metasomatized mantle peridotite with implications for deep Earth volatile cycles. *J. Geophys. Res. Solid Earth* **129**, e2023JB028202 (2024).

53. D. Grassi, M. W. Schmidt, The melting of carbonated pelites from 70 to 700 km depth. *J. Petrol.* **52**, 765–789 (2011).

54. F. Horton, Rapid recycling of subducted sedimentary carbon revealed by Afghanistan carbonatite volcano. *Nat. Geosci.* **14**, 1–5 (2021).

55. E. S. Kiseeva, G. M. Yaxley, J. Hermann, K. D. Litasov, A. Rosenthal, V. S. Kamenetsky, An experimental study of carbonated eclogite at 3·5-5·5 GPa—Implications for silicate and carbonate metasomatism in the cratonic mantle. *J. Petrol.* **53**, 727–759 (2012).

56. C. Gerbode, R. Dasgupta, Carbonate-fluxed melting of MORB-like pyroxenite at 2·9 GPa and genesis of HIMU ocean Island basalts. *J. Petrol.* **51**, 2067–2088 (2010).

57. R. Dasgupta, M. M. Hirschmann, N. D. Smith, Partial melting experiments of peridotite + CO_2 at 3 GPa and genesis of alkalic ocean island basalts. *J. Petrol.* **48**, 2093–2124 (2007).

58. A. Mallik, R. Dasgupta, Reactive infiltration of MORB-Eclogite-derived carbonated silicate melt into fertile peridotite at 3GPa and genesis of alkalic magmas. *J. Petrol.* **54**, 2267–2300 (2013).

59. M. Parolari, A. Gómez-Tuena, C. Errázuriz-Henao, J. G. Cavazos-Tovar, Orogenic andesites and their link to the continental rock cycle. *Lithos* **382–383**, 105958 (2021).

60. H. R. Marschall, J. C. Schumacher, Arc magmas sourced from mélange diapirs in subduction zones. *Nat. Geosci.* **5**, 862–867 (2012).

61. P. H. Barry, J. M. de Moor, D. Giovannelli, M. Schrenk, D. R. Hummer, T. Lopez, C. A. Pratt, Y. A. Segura, A. Battaglia, P. Beaudry, G. Bini, M. Cascante, G. d'Errico, M. di Carlo, D. Fattorini, K. Fullerton, E. Gazel, G. González, S. A. Halldórsson, K. Iacovino, J. T. Kulongoski, E. Manini, M. Martínez, H. Miller, M. Nakagawa, S. Ono, S. Patwardhan, C. J. Ramírez, F. Regoli, F. Smedile, S. Turner, C. Vetriani, M. Yücel, C. J. Ballentine, T. P. Fischer, D. R. Hilton, K. G. Lloyd, Forearc carbon sink reduces long-term volatile recycling into the mantle. *Nature* **568**, 487–492 (2019).

62. B. Z. Klein, M. D. Behn, On the evolution and fate of sediment diapirs in subduction zones. *Geochem. Geophys. Geosyst.* **22**, e2021GC009873 (2021).

63. B. L. Cousens, J. F. Allan, M. P. Gorton, Subduction-modified pelagic sediments as the enriched component in back-arc basalts from the Japan Sea: Ocean drilling program sites 797 and 794. *Contrib. Mineral. Petrol.* **117**, 421–434 (1994).

64. K. Tsuno, R. Dasgupta, Melting phase relation of nominally anhydrous, carbonated pelitic-eclogite at 2.5-3.0 GPa and deep cycling of sedimentary carbon. *Contrib. Mineral. Petrol.* **161**, 743–763 (2011).

65. S. Skora, J. D. Blundy, R. A. Brooker, E. C. R. Green, J. C. M. de Hoog, J. A. D. Connolly, Hydrous phase relations and trace element partitioning behaviour in calcareous sediments at subduction-zone conditions. *J. Petrol.* **56**, 953–980 (2015).

66. A. Fitzpayne, A. Giuliani, J. Hergt, D. Phillips, P. Janney, New geochemical constraints on the origins of MARID and PIC rocks: Implications for mantle metasomatism and mantle-derived potassic magmatism. *Lithos* **318–319**, 478–493 (2018).

67. G. L. Zhang, L. H. Chen, M. G. Jackson, A. W. Hofmann, Evolution of carbonated melt to alkali basalt in the South China Sea. *Nat. Geosci.* **10**, 229–235 (2017).

68. M. B. Baker, P. J. Wyllie, High-pressure apatite solubility in carbonate-rich liquids: Implications for mantle metasomatism. *Geochim. Cosmochim. Acta* **56**, 3409–3422 (1992).

69. T. Pausch, B. Joachim-Mrosko, A. C. Withers, T. Ludwig, J. Vazhakuttiyakam, J. Konzett, The role of calcium phosphates and silicates in the storage and transport of phosphorus at the upper-to-lower mantle transition: An experimental study to 25 GPa in a model peridotitic bulk composition. *Geochim. Cosmochim. Acta* **374**, 200–216 (2024).

70. J. Hermann, D. Rubatto, Accessory phase control on the trace element signature of sediment melts in subduction zones. *Chem. Geol.* **265**, 512–526 (2009).

71. T. Hammouda, J. Chantel, J. L. Devidal, Apatite solubility in carbonatitic liquids and trace element partitioning between apatite and carbonatite at high pressure. *Geochim. Cosmochim. Acta* **74**, 7220–7235 (2010).

72. G. M. Yaxley, M. Anenburg, S. Tappe, S. Decree, T. Guzmics, Carbonatites: Classification, sources, evolution, and emplacement. *Annu. Rev. Earth Planet. Sci.* **50**, 261–293 (2022).

73. E. B. Watson, Apatite and phosphorus in mantle source regions: An experimental study of apatite/melt equilibria at pressures to 25 kbar. *Earth Planet. Sci. Lett.* **51**, 322–335 (1980).

74. J. M. D. Day, F. Moynier, O. Ishizuka, A partial melting control on the Zn isotope composition of basalts. *Geochem. Perspect. Lett.* **23**, 11–16 (2022).

75. J. Huang, S. Chen, X. Zhang, F. Huang, Effects of melt percolation on Zn isotope heterogeneity in the mantle: Constraints from peridotite massifs in Ivrea-Verbano Zone, Italian alps. *J. Geophys. Res. Solid Earth* **123**, 2706–2722 (2018).

76. S. A. Liu, Z. Z. Wang, C. Yang, S. G. Li, S. Ke, Mg and Zn isotope evidence for two types of mantle metasomatism and deep recycling of magnesium carbonates. *J. Geophys. Res. Solid Earth* **125**, 1–22 (2020).

77. G. M. Yaxley, S. Ghosh, E. S. Kiseeva, A. Mallik, C. Spandler, A. R. Thomson, M. J. Walter, "CO_2-rich melts in Earth" in *Deep Carbon: Past to Present*, B. N. Orcutt, I. Daniel, R. Dasgupta, Eds. (Cambridge Univ. Press, 2019), pp. 129–162.

78. M. Ducher, M. Blanchard, E. Balan, Equilibrium zinc isotope fractionation in Zn-bearing minerals from first-principles calculations. *Chem. Geol.* **443**, 87–96 (2016).

79. D. Ionov, R. E. Harmer, Trace element distribution in calcite-dolomite carbonatites from spitskop: Inferences for differentiation of carbonatite magmas and the origin of carbonates in mantle xenoliths. *Earth Planet. Sci. Lett.* **198**, 495–510 (2002).

80. T. Plank, C. Langmuir, The chemical composition of subducting sediment and its consequences for the crust and mantle. *Chem. Geol.* **145**, 325–394 (1998).

81. M. C. Johnson, T. Plank, Dehydration and melting experiments constrain the fate of subducted sediments. *Geochem. Geophys. Geosyst.* **1**, 1007 (2000).

82. D. Grassi, M. W. Schmidt, D. Günther, Element partitioning during carbonated pelite melting at 8, 13 and 22 GPa and the sediment signature in the EM mantle components. *Earth Planet. Sci. Lett.* **327–328**, 84–96 (2012).

83. B. Marcaillou, G. Spence, K. Wang, J. Y. Collot, A. Ribodetti, Thermal segmentation along the N. Ecuador–S. Colombia margin (1–4°N): Prominent influence of sedimentation rate in the trench. *Earth Planet. Sci. Lett.* **272**, 296–308 (2008).

84. E. M. Syracuse, P. E. van Keken, G. A. Abers, D. Suetsugu, C. Bina, T. Inoue, D. Wiens, M. Jellinek, The global range of subduction zone thermal models. *Phys. Earth Planet. In.* **183**, 73–90 (2010).

85. A. Gómez-Tuena, J. G. Cavazos-Tovar, M. Parolari, S. M. Straub, R. Espinasa-Pereña, Geochronological and geochemical evidence of continental crust 'relamination' in the origin of intermediate arc magmas. *Lithos* **322**, 52–66 (2018).

86. A. Castro, T. Gerya, A. García-Casco, C. Fernández, J. Díaz-Alvarado, I. Moreno-Ventas, I. Löw, Melting relations of MORB-sediment mélanges in underplated mantle wedge plumes; Implications for the origin of Cordilleran-type batholiths. *J. Petrol.* **51**, 1267–1295 (2010).

87. M. D. Behn, P. B. Kelemen, G. Hirth, B. R. Hacker, H. J. Massonne, Diapirs as the source of the sediment signature in arc lavas. *Nat. Geosci.* **4**, 641–646 (2011).

88. M. L. Gorring, S. M. Kay, Mantle processes and sources of neogene slab window magmas from Southern Patagonia, Argentina. *J. Petrol.* **42**, 1067–1094 (2001).

89. C. Biellmann, P. Gillet, F. Guyot, J. Peyronneau, B. Reynard, Experimental evidence for carbonate stability in the Earth's lower mantle. *Earth Planet. Sci. Lett.* **118**, 31–41 (1993).

90. M. Hong, L. Dai, H. Hu, X. Zhang, C. Li, High-temperature and high-pressure phase transition of natural barite investigated by raman spectroscopy and electrical conductivity. *Front. Earth Sci.* **10**, 864183 (2022).

91. L. Wang, T. M. Kusky, A. Polat, S. Wang, X. Jiang, K. Zong, J. Wang, H. Deng, J. Fu, Partial melting of deeply subducted eclogite from the Sulu orogen in China. *Nat. Commun.* **5**, 5604 (2014).

92. S. G. Nielsen, T. J. Horner, H. V. Pryer, J. Blusztajn, Y. Shu, M. D. Kurz, V. Le Roux, Barium isotope evidence for pervasive sediment recycling in the upper mantle. *Sci. Adv.* **4**, eaas8675 (2018).

93. Z.-T. Shu, S.-A. Liu, D. Prelevi, Recycling of carbonates into the deep mantle beneath central Balkan Peninsula: Mg-Zn isotope evidence. *Lithos* **433**, 106899 (2022).

94. M. L. Li, S. A. Liu, H. Y. Lee, C. Yang, Z. Z. Wang, Magnesium and zinc isotopic anomaly of Cenozoic lavas in central Myanmar: Origins and implications for deep carbon recycling. *Lithos* **386–387**, 106011 (2021).

95. A. Gómez-Tuena, B. Díaz-Bravo, A. Vázquez-Duarte, O. Pérez-Arvizu, L. Mori, Andesite petrogenesis by slab-derived plume pollution of a continental rift. *Geol. Soc. Lond. Spec. Publ.* **385**, 65–101 (2014).

96. S. M. Straub, A. Gómez-Tuena, I. N. Bindeman, L. L. Bolge, P. A. Brandl, R. Espinasa-Perena, L. Solari, F. M. Stuart, P. Vannucchi, G. F. Zellmer, Crustal recycling by subduction erosion in the central Mexican Volcanic Belt. *Geochim. Cosmochim. Acta* **166**, 29–52 (2015).

97. T. Tanaka, S. Togashi, H. Kamioka, H. Amakawa, H. Kagami, T. Hamamoto, M. Yuhara, Y. Orihashi, S. Yoneda, H. Shimizu, T. Kunimaru, K. Takahashi, T. Yanagi, T. Nakano, H. Fujimaki, R. Shinjo, Y. Asahara, M. Tanimizu, C. Dragusanu, JNdi-1: A neodymium isotopic reference in consistency with LaJolla neodymium. *Chem. Geol.* **168**, 279–281 (2000).

98. W. Todt, R. A. Cliff, A. Hanser, A. W. Hofmann, "Evaluation of a ^{202}Pb-^{205}Pb double spike for high-precision lead isotope analysis" in *Earth Processes: Reading the Isotopic Code*, Geophysical Monograph Series 95, A. R. Basu, S. R. Hart, Eds., (American Geophysical Union, 1996), pp. 429–437.

99. F. Moynier, M. Le Borgne, High precision zinc isotopic measurements applied to mouse organs. *J. Vis. Exp.* **99**, e52479 (2015).

100. F. Moynier, F. Albarède, G. F. Herzog, Isotopic composition of zinc, copper, and iron in lunar samples. *Geochim. Cosmochim. Acta* **70**, 6103–6117 (2006).

101. G. P. Hayes, G. L. Moore, D. E. Portner, M. Hearne, H. Flamme, M. Furtney, G. M. Smoczyk, Slab2, a comprehensive subduction zone geometry model. *Science* **362**, 58–61 (2018).

102. F. Gervasoni, S. Klemme, A. Rohrbach, T. Grützner, J. Berndt, Experimental constraints on mantle metasomatism caused by silicate and carbonate melts. *Lithos* **282–283**, 173–186 (2017).

103. K. Hirose, I. Kushiro, Partial melting of dry peridotites at high pressures: Determination of compositions of melts segregated from peridotite using aggregates of diamond. *Earth Planet. Sci. Lett.* **114**, 477–489 (1993).

104. W. F. McDonough, S. s. Sun, The composition of the Earth. *Chem. Geol.* **120**, 223–253 (1995).

105. T. B. Thomsen, M. W. Schmidt, Melting of carbonated pelites at 2.5–5.0 GPa, silicate–carbonatite liquid immiscibility, and potassium–carbon metasomatism of the mantle. *Earth Planet. Sci. Lett.* **267**, 17–31 (2008).

106. S. Prowatke, S. Klemme, Trace element partitioning between apatite and silicate melts. *Geochim. Cosmochim. Acta* **70**, 4513–4527 (2006).

107. M. Parolari, A. Gómez-Tuena, J. G. Cavazos-Tovar, G. Hernández-Quevedo, A balancing act of crust creation and destruction along the western Mexican convergent margin. *Geology* **46**, 455–458 (2018).

108. J. Woodhead, S. Eggins, J. Gamble, High field strength and transition element systematics in island arc and back-arc basin basalts: Evidence for multi-phase melt extraction and a depleted mantle wedge. *Earth Planet. Sci. Lett.* **114**, 491–504 (1993).

109. S. M. Straub, V. Batanova, A. Sobolev, A. Gómez-Tuena, R. Espinasa-Perena, W. L. Fleming, I. N. Bindeman, F. M. Stuart, E. Widom, Y. Iizuka, The systematics of olivine CaO + Cr-spinel in high-Mg# arc volcanic rocks: Evidence for in-situ mantle wedge depletion at the arc volcanic front. *J. Petrol.* **64**, 1–19 (2023).

110. P. Condamine, S. Couzinié, A. Fabbrizio, J. L. Devidal, E. Médard, Trace element partitioning during incipient melting of phlogopite-peridotite in the spinel and garnet stability fields. *Geochim. Cosmochim. Acta* **327**, 53–78 (2022).

111. J. Adam, T. Green, Trace element partitioning between mica- and amphibole-bearing garnet lherzolite and hydrous basanitic melt: 1. Experimental results and the investigation of controls on partitioning behaviour. *Contrib. Mineral. Petrol.* **152**, 1–17 (2006).

112. P. A. Sossi, H. S. C. O'Neill, The effect of bonding environment on iron isotope fractionation between minerals at high temperature. *Geochim. Cosmochim. Acta* **196**, 121–143 (2017).

113. A. J. McCoy-West, J. G. Fitton, M. L. Pons, E. C. Inglis, H. M. Williams, The Fe and Zn isotope composition of deep mantle source regions: Insights from Baffin Island picrites. *Geochim. Cosmochim. Acta* **238**, 542–562 (2018).

114. S. Bin Fang, J. Huang, X. C. Zhang, D. A. Ionov, Z. F. Zhao, F. Huang, Zinc isotope fractionation in mantle rocks and minerals, and a revised δ^{66}Zn value for the Bulk Silicate Earth. *Geochim. Cosmochim. Acta* **338**, 79–92 (2022).

115. M. L. Pons, B. Debret, P. Bouilhol, A. Delacour, H. Williams, Zinc isotope evidence for sulfate-rich fluid transfer across subduction zones. *Nat. Commun.* **7**, 13794 (2016).

116. L. B. Cooper, D. M. Ruscitto, T. Plank, P. J. Wallace, E. M. Syracuse, C. E. Manning, Global variations in H_2O/Ce: 1. Slab surface temperatures beneath volcanic arcs. *Geochem. Geophys. Geosyst.* **13**, Q03024 (2012).

117. T. L. Grove, C. B. Till, E. Lev, N. Chatterjee, E. Médard, Kinematic variables and water transport control the formation and location of arc volcanoes. *Nature* **459**, 694–697 (2009).

118. D. E. LaRowe, S. Arndt, J. A. Bradley, E. R. Estes, A. Hoarfrost, S. Q. Lang, K. G. Lloyd, N. Mahmoudi, W. D. Orsi, S. R. Shah Walter, A. D. Steen, R. Zhao, The fate of organic carbon in marine sediments - New insights from recent data and analysis. *Earth. Sci. Rev.* **204**, 103146 (2020).

119. J. R. Cann, "Sites 501 and 504: Sediments and ocean crust in an area of high heat flow on the southern flank of the Costa Rica Rift (DSDP)," in *Initial Reports of the DSDP, vol. 69, Puntarenas to Balboa* (U.S. Government Printing Office, 1983), vol. 68, 31–173.

120. J. D. Hays, H. E. Cook, J. W. Johnson III, C. D. Hollister, F. M. Gradstein, G. T. Tucholke, M. R. Moberly, E. D. Olson, "Site 84," in *Initial Reports of the Deep Sea Drilling Project* (U.S. Government Printing Office, 1972), vol. 9.

121. M. J. Le Bas, Nephelinitic and basanitic rocks. *J. Petrol.* **30**, 1299–1312 (1989).

122. V. J. M. Salters, A. Stracke, Composition of the depleted mantle. *Geochem. Geophys. Geosyst.* **5**, Q05B07 (2004).

123. B. H. Chilson-Parks, F. M. Calabozo, A. E. Saal, Z. Wang, S. Mallick, I. A. Petrinovic, F. A. Frey, The signature of metasomatized subcontinental lithospheric mantle in the basaltic magmatism of the Payenia Volcanic Province, Argentina. *Geochem. Geophys. Geosyst.* **23**, e2021GC010071 (2022).

124. F. Moynier, D. Vance, T. Fujii, P. Savage, The isotope geochemistry of zinc and copper. *Rev. Mineral. Geochem.* **82**, 543–600 (2017).
